# Recent advances in two-dimensional nanomaterials for sustainable wearable electronic devices

**DOI:** 10.1186/s12951-023-02274-7

**Published:** 2024-02-15

**Authors:** Jing Hu, Mingdong Dong

**Affiliations:** 1 Interdisciplinary Nanoscience Center, Aarhus University, 8000 Aarhus C, Denmark; 2 Hangzhou Global Scientific and Technological Innovation Center, Zhejiang University, Hangzhou, 311215 China

**Keywords:** Two-dimensional nanomaterials, Wearable electronics, Flexible sensors, Energy devices

## Abstract

The widespread adoption of smart terminals has significantly boosted the market potential for wearable electronic devices. Two-dimensional (2D) nanomaterials show great promise for flexible, wearable electronics of next-generation electronic materials and have potential in energy, optoelectronics, and electronics. First, this review focuses on the importance of functionalization/defects in 2D nanomaterials, a discussion of different kinds of 2D materials for wearable devices, and the overall structure–property relationship of 2D materials. Then, in this comprehensive review, we delve into the burgeoning realm of emerging applications for 2D nanomaterial-based flexible wearable electronics, spanning diverse domains such as energy, medical health, and displays. A meticulous exploration is presented, elucidating the intricate processes involved in tailoring material properties for specific applications. Each research direction is dissected, offering insightful perspectives and dialectical evaluations that illuminate future trajectories and inspire fruitful investigations in this rapidly evolving field.

**Graphical Abstract:**

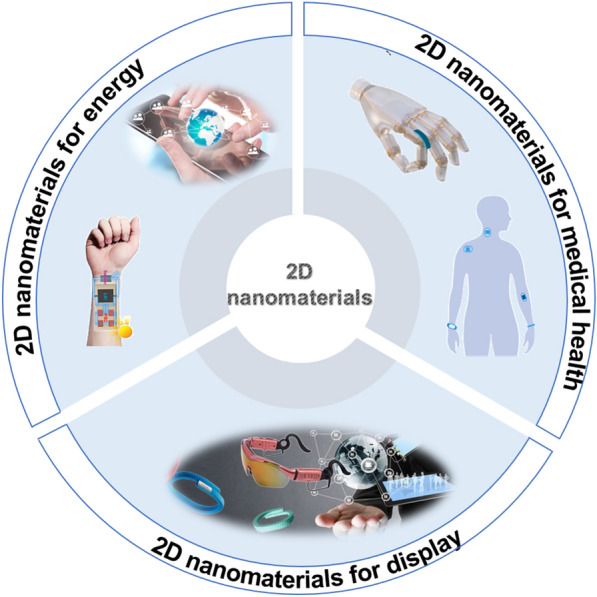

## Introduction

Flexible and wearable electronics are composed of functional parts, display parts and binding parts, which have the characteristics of wearability, portability and intelligence to perform the specific functions [1, 2]. During the past few decades, with the rise of advanced technologies such as the Internet, supercomputing, big data, and brain science, human beings have entered the era of intelligence [3]. Flexible wearable devices have found extensive applications in detecting human daily activities, monitoring health, diagnosing therapies, and supporting clinical treatments [4–6]. A wearable sensor normally consists of three basic parts: a flexible support substrate, a sensing element, and a signal output unit [7]. Among them, sensing elements have a pivotal effect on determining the performance and effectiveness of flexible wearable electronics [8]. The development of multimodal sensors that can simultaneously monitor multiple signals and integrate with self-powered systems, wireless transmission systems, etc. will facilitate the widespread use of wearable sensors in personal health management and home-based diagnosis.

Nanomaterials, which can effectively enhance the contact area between sensors and target molecules. This enhancement significantly improves sensor detection sensitivity, making them invaluable for flexible wearable sensors widely utilized in various applications [9]. Currently, a wide array of nanomaterials, ranging from zero-dimensional particles to one-dimensional, two-dimensional (2D), and three-dimensional (3D) composite structures, are currently being employed to develop advanced sensors [10]. Among them, 2D layered materials have an atomically thin planar structure, excellent mechanical flexibility and electrical properties, offer a multitude of surface-active sites, which can perform high-sensitivity and selective response to specific analytes [11, 12]. 2D nanomaterials encompass a diverse group of substances characterized by their atomic layer thickness, signifying that their dimensions are minimized to the extreme in one direction while remaining relatively large in the other two dimensions [13]. It is ideal for building flexible and wearable sensors [14]. With the continuous development of 2D materials, composite materials with heterogeneous structures can be created by doping various nanomaterials or molecules to further increase the ability of sensors. In addition, flexible wearable sensors with 2D materials are easier to assemble with systems such as self-power supply, wireless transportation, and therapeutic feedback to build integrated monitoring and therapeutic systems [2, 15, 16].

Graphene holds the distinction of being the most common and extensively validated 2D material. Novoselov and colleagues reported the advanced 2D graphene, obtained through mechanical exfoliation from highly oriented cracked graphite, showcasing its distinctive and outstanding electrical properties [17]. Since then, 2D materials, led by graphene, have experienced rapid development, giving rise to various new materials. The quantum confinement effect in atomic layer imparts unique properties to these 2D materials, setting them apart from their 3D counterparts. Consequently, they have garnered significant interest from both scientific and industrial communities. Apart from graphene, there exists a wide array of 2D materials including: single-element silicene, germanene, stannene, boronene, black phosphorus, transition metal chalcogenides, main group metal chalcogenides, etc [18]. Indeed, these 2D materials exhibit vastly diverse energy band structures and electrical properties, encompassing a wide spectrum from superconductors, semiconductors to insulators. Simultaneously, they boast outstanding optical, mechanical, thermal, and magnetic properties. By strategically stacking different types of 2D materials, it’s possible to create highly functional material systems. This capability is anticipated to drive applications in high-performance electronic, optoelectronic devices, as well as in energy conversion and storage technologies [19].

**Scheme 1:**
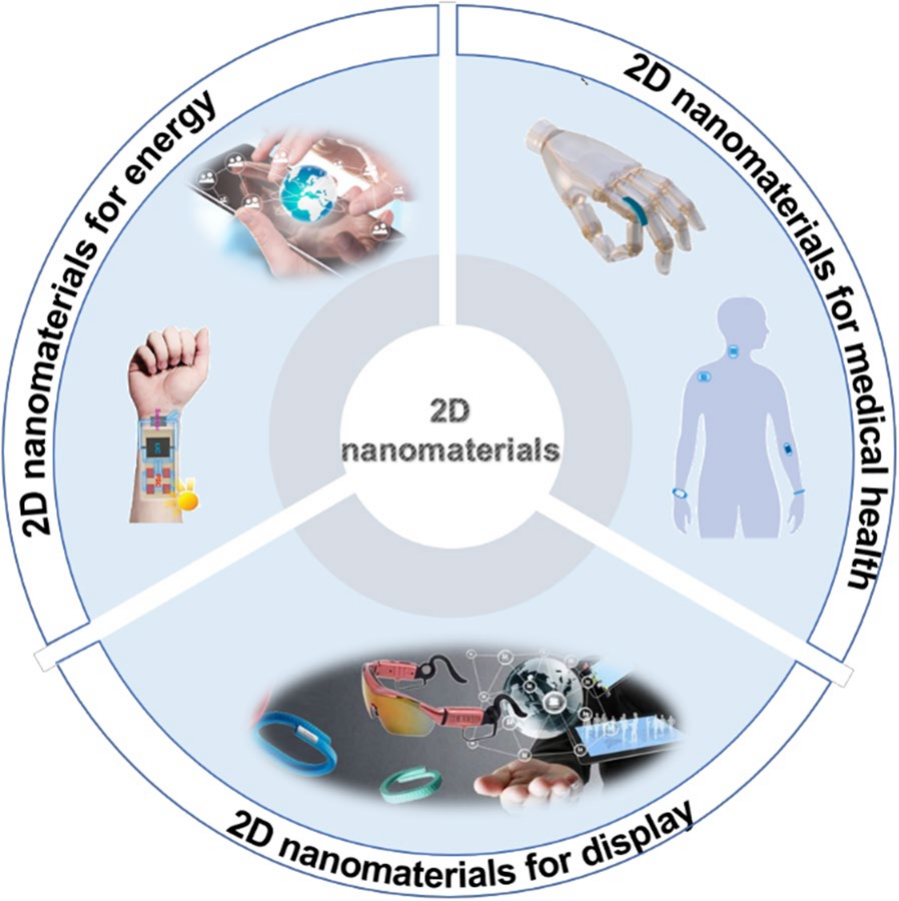
A brief introduction of two-dimensional material-based sustainable wearable energy devices. Reproduced with permission [20]. Copyright 2022, Springer Nature. Reproduced with permission [21]. Copyright 2021, Elsevier Ltd

This review first introduces the latest research progress of flexible wearable sensor devices using 2D materials, and displays a comprehensive overview of the diverse applications of 2D materials in wearable devices. Second, we summarize and analyze the integrated system based on 2D material composite flexible wearable sensors. Finally, the review concludes by discussing the existing challenges and prospects for future development of flexible wearable sensors utilizing 2D materials, providing valuable insights and guidance for the research direction of wearable sensors (Scheme 1) [20, 21].

## 2D nanomaterials

### Graphene

**Fig. 1:**
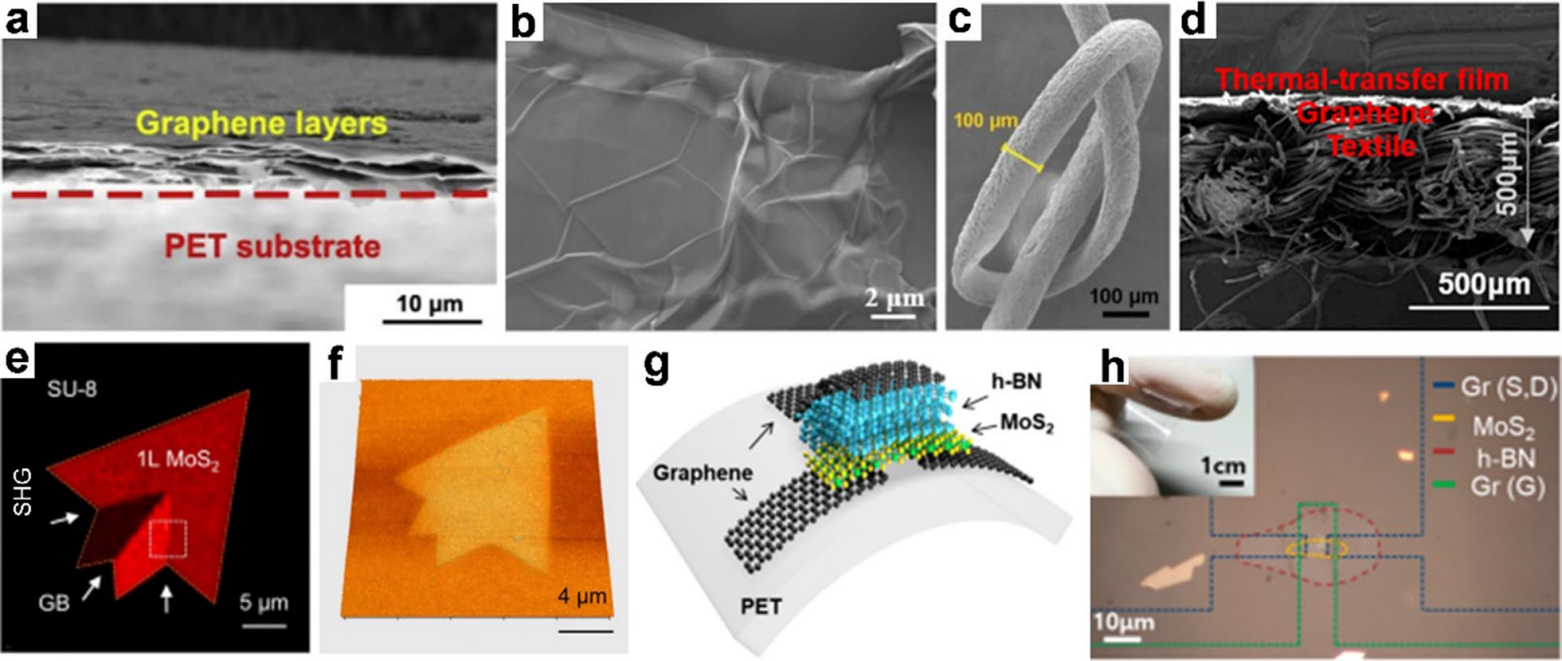
Graphene-based and MoS_2_-based multifunctional textiles. **a** The structure of prototype pressure sensor arrays created using a rGO electrode and a PET substrate. **b** A wearable humidity sensor designed around a porous graphene network. **c** Fibers made of polymers with nanoball-decorated graphene integrated into the composition. **d** The morphology of graphene integrated into the textile material. **e** and **f** Changes in the morphology and structure of isolated monolayer molybdenum disulfide (MoS_2_) crystals, observed at different dissolution time. **g**, **h** A flexible and transparent graphene/MoS_2_, depicted in both schematic and optical forms. **a** Reproduced with permission [24]. Copyright 2016, Elsevier Ltd. **b** Reproduced with permission [25]. Copyright 2018, Elsevier B.V. **c** Reproduced with permission [27]. Copyright 2019, John Wiley and Sons. **d** Reproduced with permission [26]. Copyright 2021, American Chemical Society. **e**, **f** Reproduced with permission [28]. Copyright 2018, Springer Nature. **g**, **h** Reproduced with permission [29] Copyright 2019, American Chemical Society

Graphene, characterized by its honeycomb lattice of carbon atoms, exhibits remarkable electron transport capabilities, thermal conductivity, mechanical strength, and biocompatibility [22]. Graphene-based flexible wearable sensors have been employed to detect a wide array of physical, chemical and physiological parameters, including pressure, temperature, pH value, cell, DNA, protein and other signals, and have found widespread application in flexible electronics and wearable devices [23]. Wan et al. used reduced graphene oxide (rGO) and graphene oxide (GO) to develop an all-graphene capacitive pressure sensor (Fig. 1a). The application of a slight external pressure causes the distance between the upper and lower electrodes to decrease. This change leads to an increase in capacitance, enabling the conversion between pressure and capacitance. The sensor can quickly respond to the external pressure of 0.24 Pa, and achieves an impressive pressure sensitivity of 0.8 kPa^−1^ even under minimal pressure conditions [24]. Single-layer graphene has a zero bandgap, which limits its response time and photoelectric efficiency for use in sensors. To improve its electrical properties, surface engineering can be used to introduce functional groups into graphene, thus expanding its potential applications in a variety of fields. Pang et al. innovatively crafted an unique graphene network structure using chemical vapor deposition and chemical etching techniques. The authors utilized this structure to create a highly efficient humidity sensor tailored for applications in human healthcare and activity monitoring (Fig. 1b) [25].

Graphene-based multifunctional textiles designed for sensing applications are considered ideal devices for health monitoring. Porous fibers, consisting of graphene and adorned with nanoballs, were meticulously crafted using an extended phase-separation process. This method significantly reduced the interconnection between graphene sheets and polymers by minimizing the contact area, as illustrated in Fig. 1c. The designed structure bestowed the graphene fibers with remarkably high gauge factors, measuring 51 in the 0–5% strain range and 87 in the 5–8% strain range. These values were nearly 10 times larger than similar structures lacking nanoballs. Furthermore, the graphene fibers exhibited a detection of 0.01% strain and remarkable durability, enduring 6000 cycling without deterioration. Due to its cost-effectiveness and swift fabrication process, combined with its outstanding performance and flexibility, the developed sensor exhibits significant potential in the realm of future wearable electronics. Ren et al. also contributed to this field by creating a wearable graphene-based textile utilizing advanced techniques. Graphene was meticulously transferred onto the textile, forming a sandwich-like structure comprising the textile, graphene, and thermal-transfer film, as depicted in Fig. 1d. This innovative design resulted in a composite. The graphene-based multifunctional textile exhibited an impressive coefficient of determination, reaching as high as 0.993. The graphene-based multifunctional textile demonstrated remarkable stability under continuous pressure, withstanding up to 1000 kPa, and exhibited rapid responsiveness with a quick response time of approximately 85 ms at 4.2 Pa for pressure sensing. When utilized as a physiological electric sensor, graphene-based multifunctional textile showed good detection capability when measuring the signals. Furthermore, this textile can emit specific warning sounds for health monitoring relying on the thermoacoustic effect rather than relying on mechanical vibrations. In summary, this highly linear and multifunctional integrated graphene-based textile, with its compatibility in the manufacturing process, is anticipated to significantly enhance the practical applications of in situ health monitoring [26].

### Transition metal dichalcogenides (TMDs)

Transition metal dichalcogenides (TMDs) are typically denoted by the chemical formula MX_2_, where M signifies a transition metal, and X represents a chalcogen atom [30]. Examples of TMDs include molybdenum disulfide (MoS_2_), vanadium disulfide (VS_2_), tungsten disulfide (WS_2_), tungsten diselenide (WSe_2_), and similar compounds [31]. TMDs have a wide band gap greater than 1 eV and excellent electrical properties, belong to the category of 2D materials frequently employed in wearable sensors [32]. Atomically thin MoS_2_ nanosheets have excellent flexibility, biocompatibility, and electrical properties, TMDs represent the most promising materials within their category and find extensive applications in the fields of physics, chemistry, and biosensors [33]. Chen et al. developed an implantable multifunctional sensor using monolayer molybdenum disulfide (MoS_2_) through an in-situ chemical vapor deposition (CVD) process in phosphate buffered saline solutions. The process involved varying temperatures and pH levels to achieve the desired sensor configuration (Fig. 1e, f). The MoS_2_-based bioabsorbable sensors created have the capability to monitor various parameters, including pressure, temperature, strain, and acceleration. In addition, the sensor is biodegradable and can be completely degraded within a few months, avoiding side effects in biomedical applications [28]. Park et al. harnessed the semiconducting and mechanical properties of MoS_2_ to create a large-area tactile sensor. This sensor exhibited an impressive pressure detection range from 1 to 120 kPa, surpassing the sensing capabilities of human skin. Moreover, it demonstrated multi-point high-sensitivity detection, allowing for precise identification of object shapes by simultaneously monitoring external pressure at multiple stages [34].

TMDs-based composite materials as substrates can improve the quantum yield of flexible wearable sensors, optimize the selectivity, and sensitivity of the sensors. Park et al. employed a selective synthesis method, utilizing laser beam annealing, to grow a WS_2_ layer on the MoS_2_ layer. They constructed a strain sensor using the WS_2_/MoS_2_ heterojunction material. Consequently, this sensor configuration proved effective in stably monitoring the movement of the human wrist [35]. Using the MoS_2_/graphene heterostructure as a foundation, Lee et al. prepared a high-performance strain sensor. When subjected to mechanical strain, the piezoelectric ion charges induced by the strain in MoS_2_ cause a shift in the Fermi level of graphene, altering the corresponding Schottky barrier in the graphene/MoS_2_ junction. This innovative design resulted in a sensor with an exceptionally high gauge factor, reaching 5.8 × 10^5^. Notably, the value is 500 times greater than conventional metal/MoS_2_ junction strain sensors, as illustrated in Fig. 1g, h [29].

### Transition metal carbides/nitrides (MXenes)

**Fig. 2:**
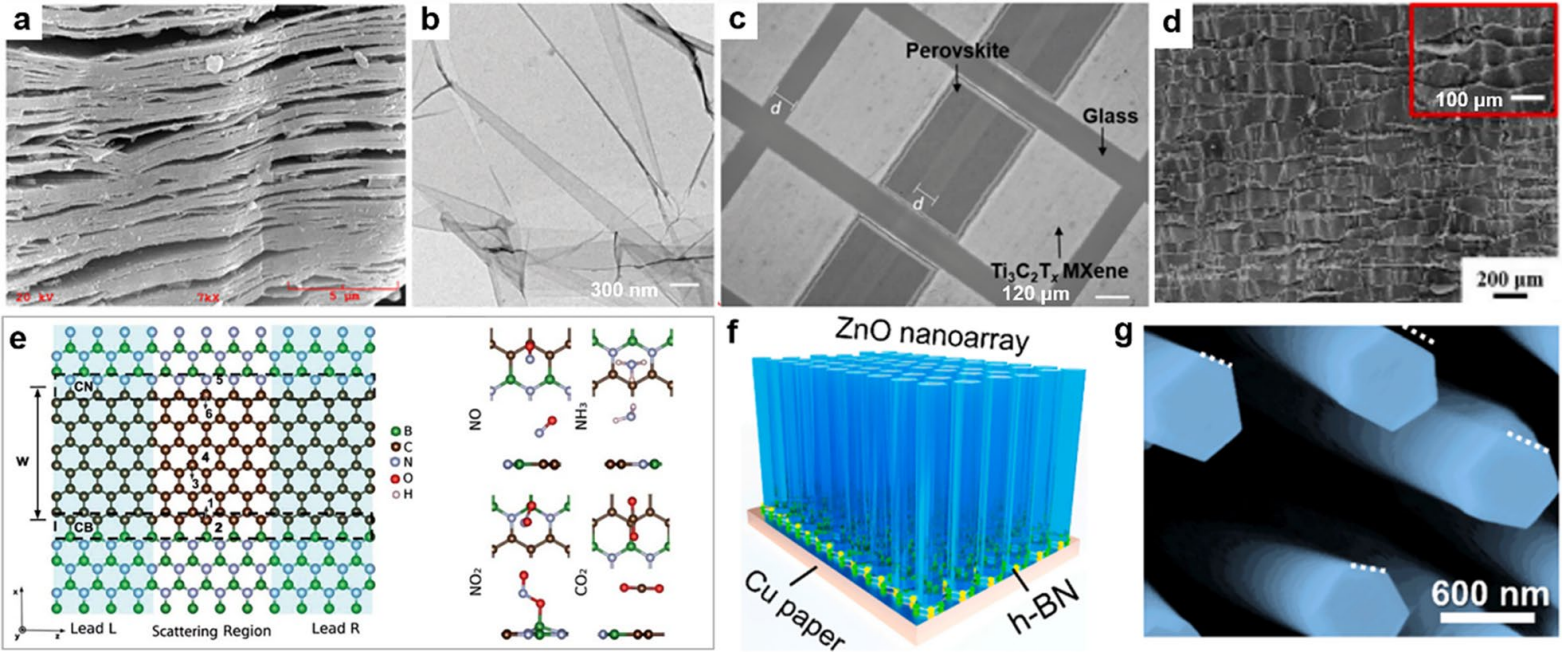
Multifunctional textiles fabricated by Mxene and other 2D nanomaterials. **a** The Ti_3_C_2_T_x_ MXene utilized for wearable sensors. **b**, **c** Ti_3_C_2_T_x_ nanosheets of MXene–perovskite image sensor arrays and the channel width of the MXene electrodes. **d** Wearable strain sensor based on MXene nanocomposites with a tile-like stacked hierarchical microstructure. **e** G-hBN sensing device corresponds to the electronic transport and illustrates the stable geometry structure. **f** and** g** Schematic representation of highly uniform ZnO nanoarray on h-BN/Cu paper and the magnifying morphology of ZnO nanoarray. **a** Reproduced with permission [39]. Copyright 2020, American Chemical Society. **b**, **c** Reproduced with permission [40]. Copyright 2020, American Chemical Society. **d** Reproduced with permission [41]. Copyright 2020, Elsevier Ltd. **e** Reproduced with permission [43]. Copyright 2019, Royal Society of Chemistry. **f**, **g** Reproduced with permission [44] Copyright 2023, Elsevier Ltd

The general formula for MXene materials is M_n+1_X_n_ or M_n+1_X_n_T_x_ (n = 1 ~ 3), where M stands for early transition metals like Ti, Zr, V, Nb, or Mo, etc., X represents C or N, and T_x_ represents surface functional groups like -OH, -O, -F, etc. [36] MXene exhibits outstanding electrical conductivity, mechanical flexibility, and chemical stability. The properties of MXene can be significantly improved through appropriate surface modification techniques, thereby expanding the potential applications of MXene in wearable sensors [37]. Recently, MXenes, a novel addition to the 2D materials family, have garnered significant interest owing to their remarkable conductivity (reaching up to ~ 10^5^ S cm^−1^), high pseudo-capacitance (up to 1600 F cm^−3^), robust mechanical properties, and excellent hydrophilicity. These characteristics position them as highly promising materials for wearable energy devices. However, creating MXene electrodes that possess optimal electrical, electrochemical, and mechanical properties simultaneously remains a significant challenge. This difficulty stems from their inherently weak interlaminar interactions and small particle size [38]. Zhang et al. introduced a self-healing Ti_3_C_2_ MXene/polydimethylsiloxane (PDMS) supramolecular elastomer, facilitated by intermolecular interactions, demonstrating exceptional mechanical strength, stability, and electrical sensitivity (Fig. 2a). The raw material utilized was D-asparagine from Liliaceae plants, modified through carboxyl and hydroxyl esterification. Additionally, 3,4-dihydroxybenzaldehyde from forest plants was grafted onto PDMS macromolecules containing amino groups via imine bonds formed through a Schiff base reaction. This Ti_3_C_2_ MXene/PDMS elastomer exhibited self-healing capabilities at room temperature, attributed to the presence of hydrogen and imine bonds. Following healing, the material's mechanical and electrical properties were almost entirely restored. The uniform dispersion of MXenes within the polymer system resulted in excellent electrical conductivity and sensitivity to changes in stress state. Notably, the mended material successfully monitored both large and small movements of human muscles [39]. Ren et al. developed a highly sensitive Mxene-based sensor for large-scale image comprising 1250 pixels. The sensor exhibited remarkable performance characteristics due to the synergistic energy level alignment and near-infrared resonance properties between Ti_3_C_2_ and perovskite. Notably, the sensor demonstrated high responsivity at 84.77 AW^−1^, specific detectivity of 3.22 × 1012 Jones, and an extensive linear dynamic range (LDR) of up to 82 dB across a broad wavelength spectrum from visible to near-infrared (Fig. 2b, c) [40].

MXene can be combined with other functional materials to leverage the synergistic advantages between these materials, resulting in the development of high-performance flexible wearable sensors. For instance, Chao et al. dispersed MXene and polyaniline fiber (PANIF) layers onto an elastic rubber substrate, creating a wearable MXene/PANIF strain sensor with a laminated structure. This strain sensor demonstrated the ability to detect a wide range of human motions (up to 80% strain) with an exceptionally low detection limit (0.1538% strain), high sensitivity [up to 2369.1 for the gauge factor (GF)], and exhibited excellent reproducibility and stability. As illustrated in Fig. 2d, the sensor can be affixed to the skin to monitor human respiration, pulse, and knuckle motion. It boasts a wide strain sensing range, an incredibly low detection limit, high sensitivity, and outstanding cycle stability [41]. Li et al. introduced a pure Ti_3_C_2_Tx MXene aerogel fiber through a straightforward dynamic sol–gel spinning process followed by supercritical CO_2_ drying. These MXene aerogel fibers showcased a unique oriented mesoporous structure and possessed electrothermal/photothermal dual-responsiveness owing to their high electrical conductivity and excellent light absorption capabilities. The amalgamation of these features positions MXene aerogel fibers as highly promising materials for applications in flexible wearable devices, smart fabrics, and portable equipment [42].

### Other 2D nanomaterials

2D materials like black phosphorus (BP) and hexagonal boron nitride (h-BN) have found applications in flexible wearable sensors [45]. BP boasts a simple manufacturing process, a large specific surface area, excellent electrical properties, and high carrier mobility. However, BP is prone to instability and oxidation when exposed to environmental oxidants, necessitating efforts to enhance its stability in sensors [46]. Introducing a passivation layer is a common method to improve BP sensor stability [47]. However, this approach often reduces sensor sensitivity, limiting BP's application in high-performance sensors. Furthermore, there are ongoing debates about the cytotoxicity and biocompatibility of BP, which restrict its use in flexible wearable sensors.

h-BN, with its excellent biocompatibility, high specific surface area, and efficient photoelectric conversion properties, stands as a key contender for wearable sensors [48]. In their work, Fabio et al. constructed a flexible sensor for the quantitative monitoring of NO and NO_2_ gas molecules, showcasing exceptional selectivity and sensitivity through the h-BN/graphene heterostructure (Fig. 2e) [43]. Additionally, Liu et al. introduced a novel approach, utilizing a 2D h-BN atomic layer film as a pre-orientation layer to achieve high orientation consistency directly on the surface of polycrystalline Cu paper (Fig. 2f, g) [44]. Leveraging the atomically smooth, dangling bond-free surface and hexagonal lattice properties of h-BN, this method holds promise for various applications. The elucidation of the nucleation, orientation, and stress release mechanisms of h-BN pre-guided ZnO nanocolumn arrays provides crucial theoretical insights and technical guidelines for the application of h-BN and other 2D materials in pre-guided layers. Furthermore, a novel h-BN/ZnO nanocolumn/h-BN sandwich structure, along with a Schottky dielectric interface, was developed. This innovation led to the creation of a flexible and transparent piezoelectric nanogenerator thin film device, a breakthrough where the dielectric layer thickness was reduced to an atomic monolayer. Remarkably, this device showcased an exceptionally high-power generation density of 169 mW cm^−2^. It was effectively employed in harvesting mechanical energy from human body movements such as walking and running, as well as in smart portable chargers. This achievement highlights the tremendous potential of flexible and transparent piezoelectric nanogenerators in the realm of future wearable self-powered electronics.

## 2D nanomaterials for sustainable wearable energy devices

### 2D nanomaterials for sensing

**Fig. 3:**
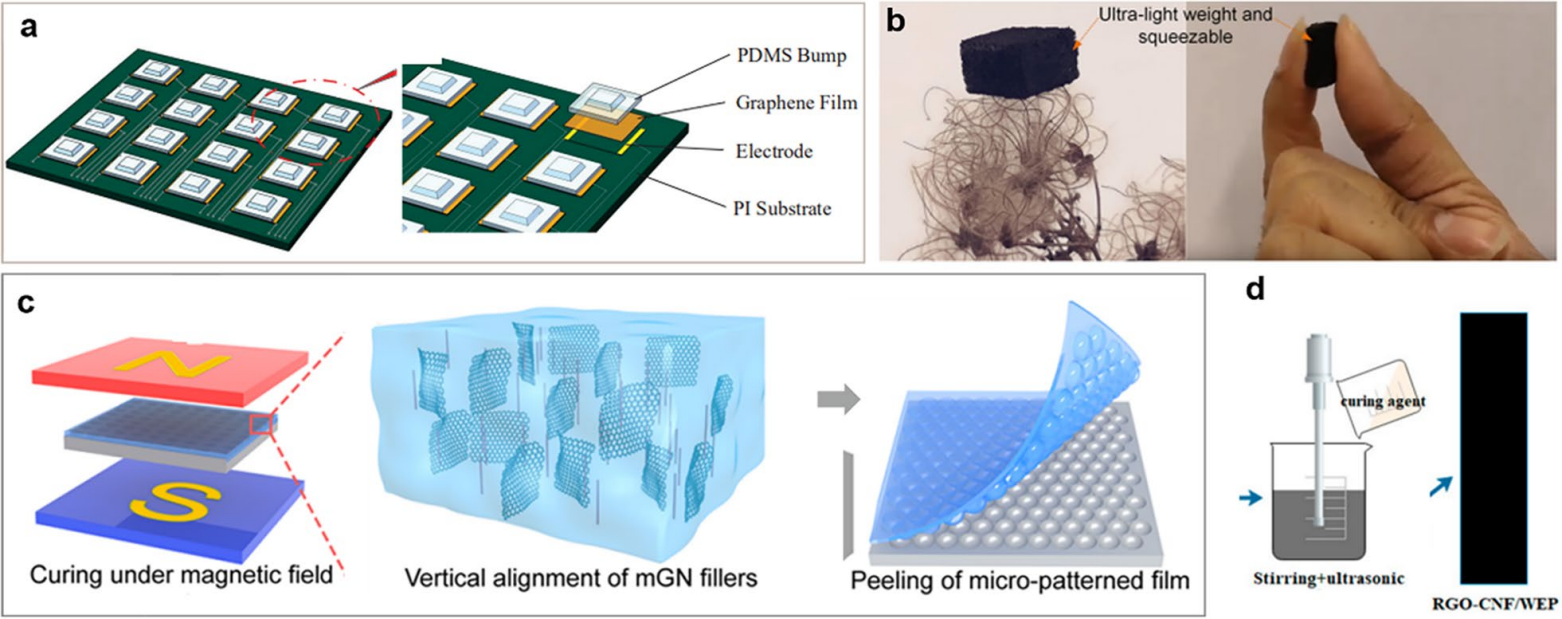
2D nanomaterials for sensing. **a** Illustration of the structure of the electronic skin. **b** 3D graphene-PDMS composite foams possess a lightweight and squeezable nature. **c** Schematic of fabricating mGN-EcoFlex film. **d** Overview of the fabrication of rGO-cellulose nanofiber hybrid filler. **a** Reproduced with permission [51]. Copyright 2019, the authors. **b** Reproduced with permission [52]. Copyright 2019, American Chemical Society. **c** Reproduced with permission [53]. Copyright 2019, American Chemical Society. **d** Reproduced with permission [54] Copyright 2019, the authors

Sensors play a pivotal role in wearable electronic devices, and the future trajectory emphasizes their miniaturization and refinement [49]. Currently, the market offers a wide array of sensors, among which inertial measurement devices, particularly accelerometers, are prevalent. Accelerometers are capable of tracking specific movement data, direction, strength, or speed of motion. For instance, in a mobile phone or tablet, when the device is rotated (input), the accelerometer processes this motion and adjusts the screen orientation accordingly (output). In the realm of sensors, 2D materials find extensive applications, particularly in electrochemical biosensors, gas sensors, and piezoresistive sensors. This prevalence is due to the unique structure and adjustable pore size of 2D materials, which enhance the detection performance of various substances. Flexible piezoresistive sensors, a vital component of wearable devices, consist of flexible substrates and conductive sensitive materials [50]. Graphene, renowned for its exceptional electrical conductivity and bending strength, finds extensive application in such sensors. Yang et al. pioneered the development of a flexible electronic skin for pressure sensing, utilizing graphene films. The image of the graphene-based sensor is illustrated in Fig. 3a. The electronic skin comprises 4 × 4 tactile sensing units, encompassing three distinct layers: the lower substrate (constructed from polyimide substrate), the pressure-resistance layer (comprising graphene/polyethylene glycol film), and the upper layer made of polydimethylsiloxane. The underlying principle of this sensor involves leveraging the pressure-resistance effect exhibited by graphene materials. When pressure is applied, the C-C bonds within the graphene film may be fractionated or broken, leading to a change in the film's resistance. Through this mechanism, the electronic skin can measure normal stress within the range of 0–500 kPa, accurately replicating the appearance of the object being sensed [51]. In addition, Sengupta et al. developed a compressible piezoresistive sensor using graphene-PDMS foam for personalized healthcare applications (Fig. 3b). Graphene nanosheets integrated into the PDMS foam's porous structure, forming a nanomaterial network. The sensor displayed remarkable stability through 36,000 cycles of compressive loading, making it suitable for human motion detection and personalized health monitoring [52].

To enhance sensor performance, graphene can be combined with other conductive sensitive materials to leverage a synergistic effect [55]. However, achieving uniform dispersion in polymers is challenging, restricting graphene's application in sensors. To overcome this challenge, graphene is often functionalized to yield graphene oxide, which contains numerous oxygen groups. This modification enhances its compatibility with other hydrophilic organic materials. Subsequently, chemical methods are used to reduce graphene oxide, improving its uniform dispersion in polymers and enabling broader applications in sensors. Wang et al. used magnetically rGO@nickel nanowire fillers as the sensing layer of flexible sensors, demonstrating a high sensitivity of 1302.1 kPa^−1^ (Fig. 3c). When self-installed at the tip of the tweezers, the sensor can monitor tube pressure at different frequencies and amplitudes [53]. Wu et al. employed rGO as a conductive filler, cellulose nanofiber (CNF) as a dispersant and structural support, and waterborne epoxy as a polymer matrix to create flexible composite sensors with a piezoresistive effect (Fig. 3d). The results revealed that the composite sensors established a stable enhanced conductive network, leading to significant changes in the mechanical properties and electrical resistivity of the composites. The resulting composite film demonstrated remarkable characteristics: it could endure substantial deformation (over 55% strain), exhibited a gauge coefficient ranging from 34 to 71, and maintained stable piezoresistive properties within a strain range of 4% [54].

**Fig. 4:**
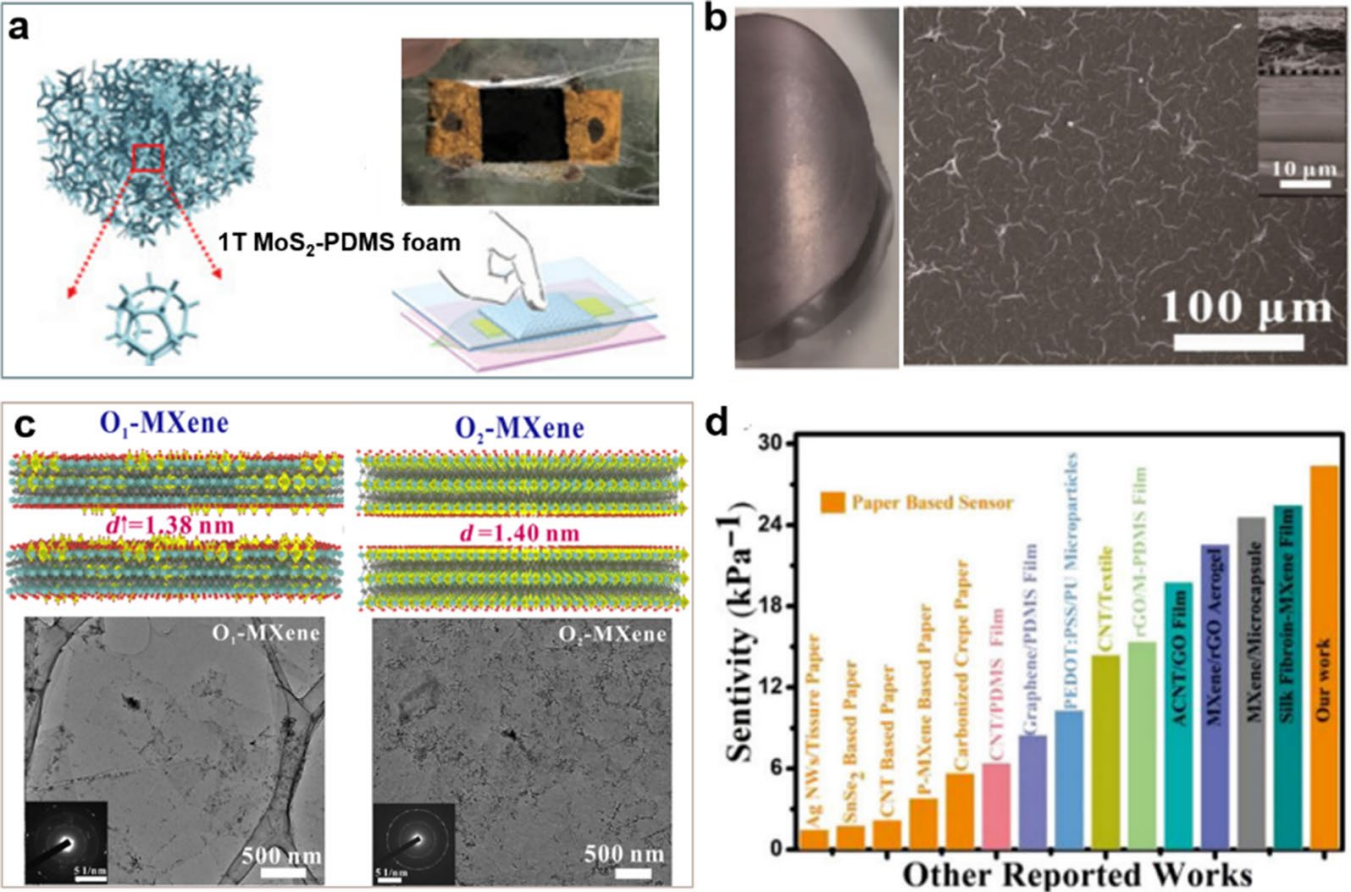
2D nanomaterials for sensing. **a** Fabrication of the 1 T MoS_2_-PDMS foam pressure sensor. **b** Structure of MXene/LDPE bilayer films. **c** The microstructure change of O-MXene sensor. **d** The performance of the pressure senor of MXene. **a** Reproduced with permission [56]. Copyright 2019, John Wiley and Sons. **b** Reproduced with permission [57]. Copyright 2021, Elsevier B.V. **c**, **d** Reproduced with permission [58] Copyright 2022, the authors

While there have been numerous studies on utilizing graphene in flexible sensors, its lack of a bandgap limits its advancement in sensor applications. However, other 2D transition metal sulfide nanomaterials (such as MoS_2_, WS_2_, etc.) have a moderate carrier mobility, which make up for the performance of graphene in this field. As a non-toxic and environmentally friendly 2D semiconductor material with tunable bandgap, MoS_2_ has been proved to have excellent performance. There are still relatively few reports on piezoresistive sensors. Yang and colleagues developed an advanced pressure sensor by incorporating MoS_2_-PDMS as a conductive active layer, along with layered micro-network veins serving as an isolation layer (Fig. 4a). The device exhibited remarkable features and demonstrate excellent stability [56].

Liu and colleagues developed a multifunctional actuator by combining MXene and low-density polyethylene through a simple drop-casting technique [59]. In this approach, the 2D MXene layer efficiently absorbs electrical energy, transforming it into thermal energy. Subsequently, this thermal energy heats the actuator, causing the thermoplastic low-density polyethylene to expand [60]. Furthermore, the actuator demonstrates the ability to function as a walking robot, achieving speeds of up to 16.52 mm min^−1^. This study introduces a groundbreaking approach to broaden MXene applications, especially in contexts like the ongoing COVID-19 situation, where non-contact solutions are essential. The actuator can serve as a light-controlled (or thermal) switch, seamlessly integrated into circuits. This innovation finds utility in applications demanding non-contact solutions, even in extreme scenarios (Fig. 4b) [57]. In recent years, researchers have extensively explored Ti3C2Tx-type MXene for constructing flexible pressure sensors known for their high sensitivity. This interest stems from MXene's metal conductivity, excellent hydrophilicity, and mechanical properties. However, during the chemical wet preparation of MXene, the transition metals’ unstable valence states lead to varying degrees of oxidation, raising questions about irreversible oxidation’s impact on electrical conductivity and sensing capabilities. Addressing this, Ma and colleagues introduced a groundbreaking paper-based MXene piezoresistive pressure sensor. Through a combination of experimental analysis and density functional theory, they delved into the impact of in-situ oxidation degree on the sensor's sensitivity. The study revealed a remarkable sensitivity for the partially oxidized MXene-based paper sensor. Additionally, MXene paper-based sensing elements outshine their polymer counterparts by being easily degradable and environmentally friendly. These MXene-based sensors exhibit promising applications (Fig. 4c, d) [58].

### 2D nanomaterials for medical health

**Fig. 5:**
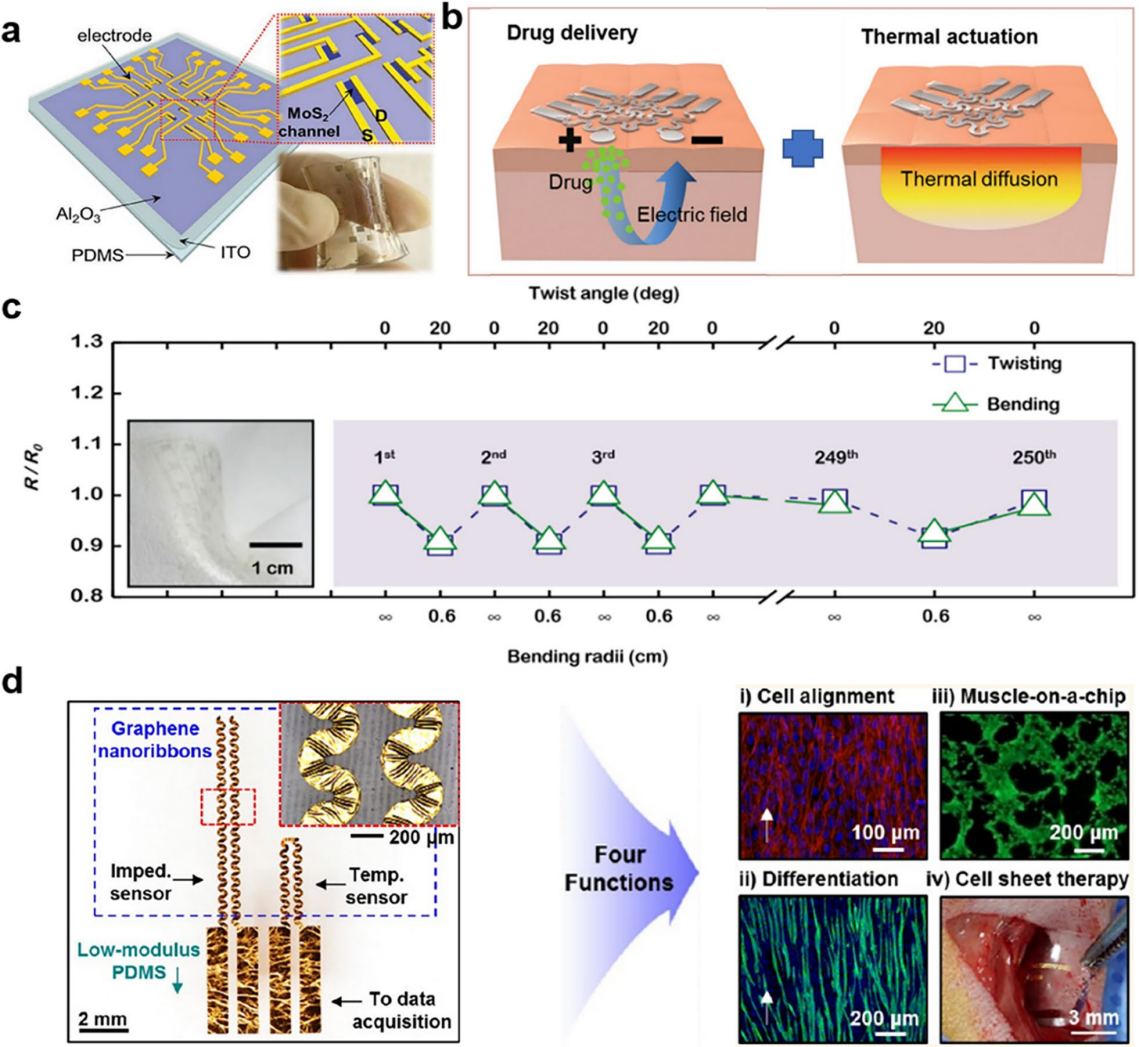
2D nanomaterials for medical health. **a** Diagram of MoS_2_ FETs array on a soft PDMS substrate with magnified MoS_2_ channels. **b** Graphene-based electronic/optoelectronic devices for transdermal drug delivery and thermal therapy. **c** Resistance changes of MoS_2_ tactile sensor during cycled experiments. **d** Versatile cell-culture platform equipped for monitoring, printing, and therapy. **a** Reproduced with permission [65]. Copyright 2017, John Wiley and Sons. **b** Reproduced with permission [67]. Copyright 2015, John Wiley and Sons. **c** Reproduced with permission [70]. Copyright 2016, John Wiley and Sons. **d** Reproduced with permission [71] Copyright 2015, American Chemical Society

The human body is mainly composed of soft, movable tissues, with neural tissue being the softest and most viscous. This mechanical mismatch between tissue organs and traditional hard, bulky medical devices can easily cause damage to neural tissue [61, 62]. To tackle this concern, soft bioelectronic devices have been engineered with mechanical properties closely resembling the human tissue. These devices can prevent unnecessary mechanical damage from implants while forming tight and robust contacts with curved and moving organs, making them suitable for long-term implantation in living organisms. 2D materials with atomic-scale thickness are ideal candidates as electronic building blocks for creating high performance bioelectronic devices [63]. For instance, graphene is able to be bent and wrinkled without altering its original electrical properties, a feat not achievable with traditional electronic materials. The reduced device thickness and increased device softness and flexibility minimize the mechanical stress of the implantable device on the target tissue. Soft biomedical devices based on 2D materials can fit perfectly to the human body without eliciting an immune response [64]. For instance, soft neural implants made from graphene exhibit remarkably low stiffness. This characteristic enables them to reduce mechanical damage to neural tissue while seamlessly integrating with the brain in a conformal manner. Ultrathin graphene-based biosensors have also been successfully implanted on curved teeth, facilitating the detection of bacterial content at the level of single-cell. Moreover, the human body releases diverse biological fluids containing vital biochemical substances that offer valuable insights into metabolism and chronic diseases. Biosensors play a pivotal role in monitoring these biochemical levels by detecting alterations in electrical signals provoked by the presence of biochemicals on the sensor surface. To achieve accurate diagnosis, these biosensors must have high sensitivity for detecting trace substances. Typically, sensitivity is contingent upon the specific surface area of the biosensor, increased surface areas resulting in larger electrical signal changes caused by the adsorption of biochemical substances. As 2D materials are atomically thin, biochemicals adsorbed by the receptors induce significantly signals than conventional bulk materials, resulting in higher sensitivity for biosensors based on 2D materials. For instance, a wearable graphene-based glucose sensor exhibits exceptional sensitivity, enabling the detection of glucose concentrations as low as 10 μM, while MoS_2_-based humidity sensors showed the sensitivity up to 10^4^ (Fig. 5a) [65]. In 2022, Bao et al. researched and designed a flexible and stretchable neurochemical biosensor based on 2D graphene, crafted through laser patterning of a metal-complexed polyimide. The sensor synergized the outstanding mechanical properties of 2D graphene with its versatile chemical sensing capabilities. This unique combination allows it to detect the dynamics of multiple neurotransmitters in both the brain and gut [66].

Absolutely, transparency is a vital characteristic for materials employed in soft bioelectronics. Opaque wearable electronics, when affixed to the skin, may lead to discomfort visualization. This potential interference emphasizes the need for using transparent or non-opaque materials in these devices to maintain clear visualization and ensure accurate surgical procedures. Fortunately, 2D materials possess atomic-level thickness, which gives them transparent characteristics, allowing most of the incident light to pass through the material rather than being absorbed (Fig. 5b) [67]. A variety of graphene-based bioelectronic devices, including, pH sensors, cell sensors, and tumor, have recently been designed and prepared [68]. Thanks to their transparency, surgeons can treat the colon cancer. Additionally, wearable graphene and MoS_2_ based strain gauges can be used to quantitatively analyze the status of daily body movements (Fig. 5c) [69, 70].

Biocompatibility is crucial for the long-cycle life wearable bioelectronic devices. Implantable devices can consistently monitor biological signals with high accuracy by measuring changes in impedance. To avoid chronic immune responses, every material that makes up a bioelectronic device should be biocompatible. 2D materials exhibit excellent biocompatibility and establish intimate contact with biological tissues (Fig. 5d) [71]. For example, graphene-based devices achieved high-quality biosensing and stimulation by building a good interface with tissues. Graphene electrodes have been combined with cell sheets, creating a biocompatible interface for physioelectric sensing. Moreover, a biodegradable sensor utilizing MoS_2_ monolayer has been developed for constructing a transient system. This MoS_2_-based biodegradable sensor is capable of monitoring intracranial strain, temperature, pressure, motion, and eventually disappears in the hydrolysis of the organism, with extremely low toxicity and no side effects [28].

### 2D nanomaterials for display

Electronic skin, an ultra-thin electronic device capable of simulating the sense of touch, stands apart from conventional hardware due to its soft, flexible nature. It can be shaped into various forms, making it adaptable for diverse applications. For instance, it can function as a coat, such as on the surface of a robot, and it can also be used in human prosthetic surgery that encounters severe skin trauma (such as burns or skin diseases). This new type of artificial skin can sense changes in external pressure, temperature, etc., and send signals to our brains through circuits, resulting in a near-real sense of touch [72]. Recently, the advancement of artificial electronic skin has achieved certain results, it must be sensitive to multiple signals such as humidity and pressure while applied to simulate, restore or even replace the skin of the body. The demand for functionally integrated device arrays has attracted more and more attention. The traditional working mode of artificial electronic skin is mostly based on contact sensing, that is, the magnitude of the mechanical stimulus can be judged by the change of the electrical signal after the external stimulus and the sensor are in contact with each other. This working mode will inevitably make the working process much slower. There is a risk of cross-infection when people operate the same equipment, especially in a pandemic device, therefore, non-contact operation mode is preferred to minimize the risk [73].

**Fig. 6:**
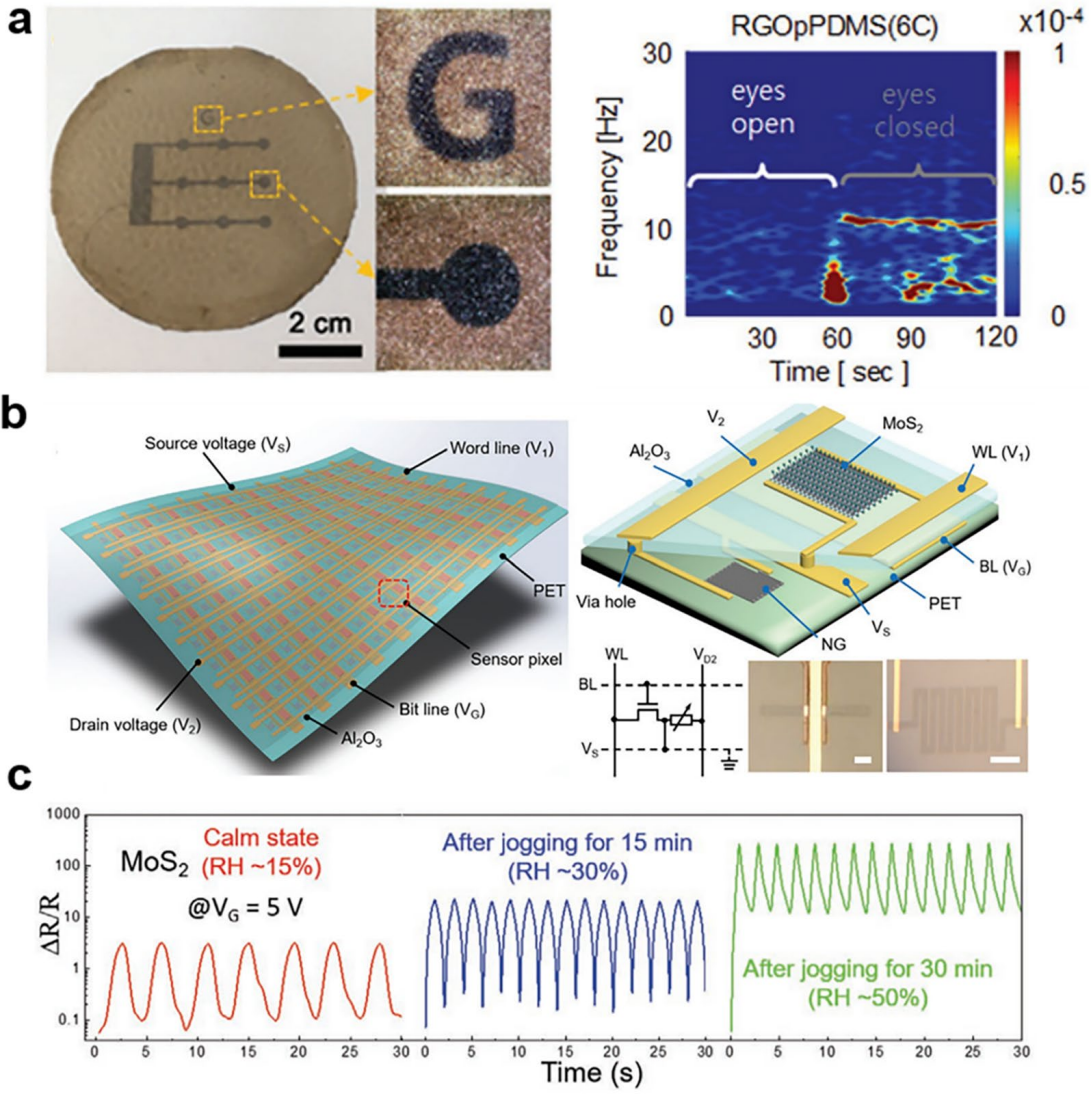
2D nanomaterials for display. **a** Porous RGO/PDMS films and devices designed to be stretchable and conductive, serving the purpose of electronic skin. **b** Illustration depicting active-matrix sensors utilizing 2D MoS_2_ materials as their foundation. **c** MoS_2_ resistance change rates indicating human respiratory humidity under various motion states. **a** Reproduced with permission [75]. Copyright 2017, John Wiley and Sons. **b**, **c** Reproduced with permission [76] Copyright 2021, John Wiley and Sons

Due to the size advantages of 2D materials and their flexibility and easy attachment to different substrates, the artificial electronic skin based on 2D nanomaterials not only has multifunctional sensing functions similar to skin, but also has high sensitivity, fast response time, multi-working Patterns and other performance far exceed human skin [74]. Professor Jun’s research team developed a multifunctional and high-performance graphene-based substrate by attaching conductive RGO sheets to flexible and porous PDMS substrate for stress-sensing electronic skin. The PDMS substrate was prepared by steam-etching and modified with 3-aminopropyltriethoxysilane and a GO coating was dip-coated, based on the introduction of Cu^2+^ as a cross-linking point to achieve GO. The controllable multi-layer construction of the sheet, and finally the selective reduction using steam, obtained different patterned RGO/porous PDMS flexible electronic sensor devices. The graphene bioelectrodes exhibited exceptional stretchability, enduring mechanical stress through 5000 cycles of stretching and releasing. Additionally, the graphene strain sensors demonstrated high sensitivity, covering a broad sensing range of up to 40% strain. The construction method of the flexible electronic skin is simple and cost-effective, eliminating the need for complex processes like vacuum sputtering, making it convenient for large-scale preparation (Fig. 6a) [75].

Zhao et al. used graphene and molybdenum disulfide devices to design a multi-modal artificial electronic skin based on comprehensive 2D materials, which enabled non-interference and high-sensitivity real-time monitoring of external strain, humidity and other signals (Fig. 6b, c). This technology was effectively employed in a human respiratory signal monitoring system, showcasing ultra-high sensitivity (over 400 for strain and approximately 104 for humidity sensing), high stability (lasting for more than 1000 cycles), and quick response. In particular, non-contact mode is proposed to provide an early warning of the position signal of an approaching object, thus effectively avoiding the risk of cross-infection when multiple people use the device in the contact mode. This achievement offers a novel concept for the further application of sensors utilizing 2D materials in artificial prosthetics, flexible smart wear and other fields, thus enabling safe and efficient use [76]. In conclusion, despite the numerous scientific issues that remain to be addressed, the future of electronic skin looks promising.

### 2D nanomaterials for energy

Capacitors, supercapacitors and batteries represented the area of energy storage devices have been widely investigated, which are designed perfectly meet the demands posed by portable/wearable electronics. 2D nanomaterials 2D nanomaterials commonly demonstrate robust covalent bonds within their plane and weaker van der Waals interactions perpendicular to the plane. They boast substantial specific surface areas and find extensive application in energy storage, thanks to their excellent electron transport, optical, thermal, and other unique properties [77]. They possess adjustable chemical and physical characteristics, making them ideal for high-performance electrochemical energy conversion and storage devices, capable of high-performance operation [78]. The quantum confinement effects and surface interactions in atomically thin 2D material nanosheets are closely tied to the atomic layers. These properties vary significantly based on the material's atomic thickness, showcasing the unique behavior of 2D nanomaterials at the atomic scale. For instance, some bulk materials are indirect bandgap semiconductors, but their monolayer nanosheets are direct bandgap semiconductors, and the number of atomic layers leads to significant changes in their properties, such as enhanced photoluminescence [79]. Unlocking 2D materials can lead to changes in a series of properties including bandgap, electrical conductivity, thermoelectric properties, photovoltaic properties and superconductivity, and to obtain new heterostructures or binding properties. The expanded interlayer distance significantly diminishes the Vander Waals interaction, emphasizing the distinctive features of single-layer characteristics. Moreover, a larger interlayer spacing results in a higher number of accessible catalytic active sites. This enhancement greatly augments the performance of 2D materials in various energy storage systems such as Li-ion batteries, Zn-ion batteries, and supercapacitors. Additionally, it benefits other energy conversion devices like solar cells and thermoelectric devices, leading to improved efficiency and functionality [2].

Rechargeable lithium-ion batteries (LIBs) represent a crucial advancement in energy storage technology, experiencing rapid development owing to their extended lifespan, high energy density [80]. Researchers have primarily concentrated on designing and synthesizing innovative electrode materials for rechargeable LIBs due to their pivotal role in determining the electrochemical performance. The focus lies in developing materials with both high lithium storage capacity and excellent conductivity [81]. 2D materials with expanded interlayers possess an open structure that enhances the storage and efficient transport of ions and electrons. This characteristic significantly enhances the performance of batteries when utilized as electrode materials for LIBs. Additionally, increasing the interlayer distance is advantageous as it reduces strain, resulting in improved efficiency of the battery system [82]. As an illustration, envision a fiber-shaped LIB intricately woven into fabric, capable of powering electrochemical analysis and facilitating wireless data transfer. Despite their prevalence in the wearable energy sector, existing lithium-ion battery technologies are predominantly planar, bulky, and rigid [83]. Zheng et al. introduced a groundbreaking innovation: the inaugural prototype of all-solid-state planar lithium-ion microcapacitors. These microcapacitors were constructed and specifically designed for flexible and wearable electronics. Employing a layer-by-layer filtration technique with graphene nanosheets exfoliated by electrochemically method, the researchers successfully created asymmetric interdigital microelectrodes featuring a planar geometry. The resulting microcapacitors exhibited remarkable flexibility, maintaining stable performance even when subjected to bending and twisting. Furthermore, they demonstrated superior integration, marking a significant advancement in the realm of flexible energy storage device [84].

Supercapacitors (SCs) emerge as a promising solution to energy storage challenges, given their high energy density, rapid charging and discharging capabilities, and stable performance [38]. To align with the evolving needs of portable electronic devices, flexible and even planar supercapacitors are developing rapidly. The 2D layered structure material becomes a potential choice. Capacitors made of 2D materials as electrode materials have good mechanical properties, not only can be curled and folded arbitrarily, but also will not cause significant performance loss. When 2D oxide materials are applied to electrolytes, almost all electrons can participate in the reaction, which has great potential in SCs [85].

**Fig. 7:**
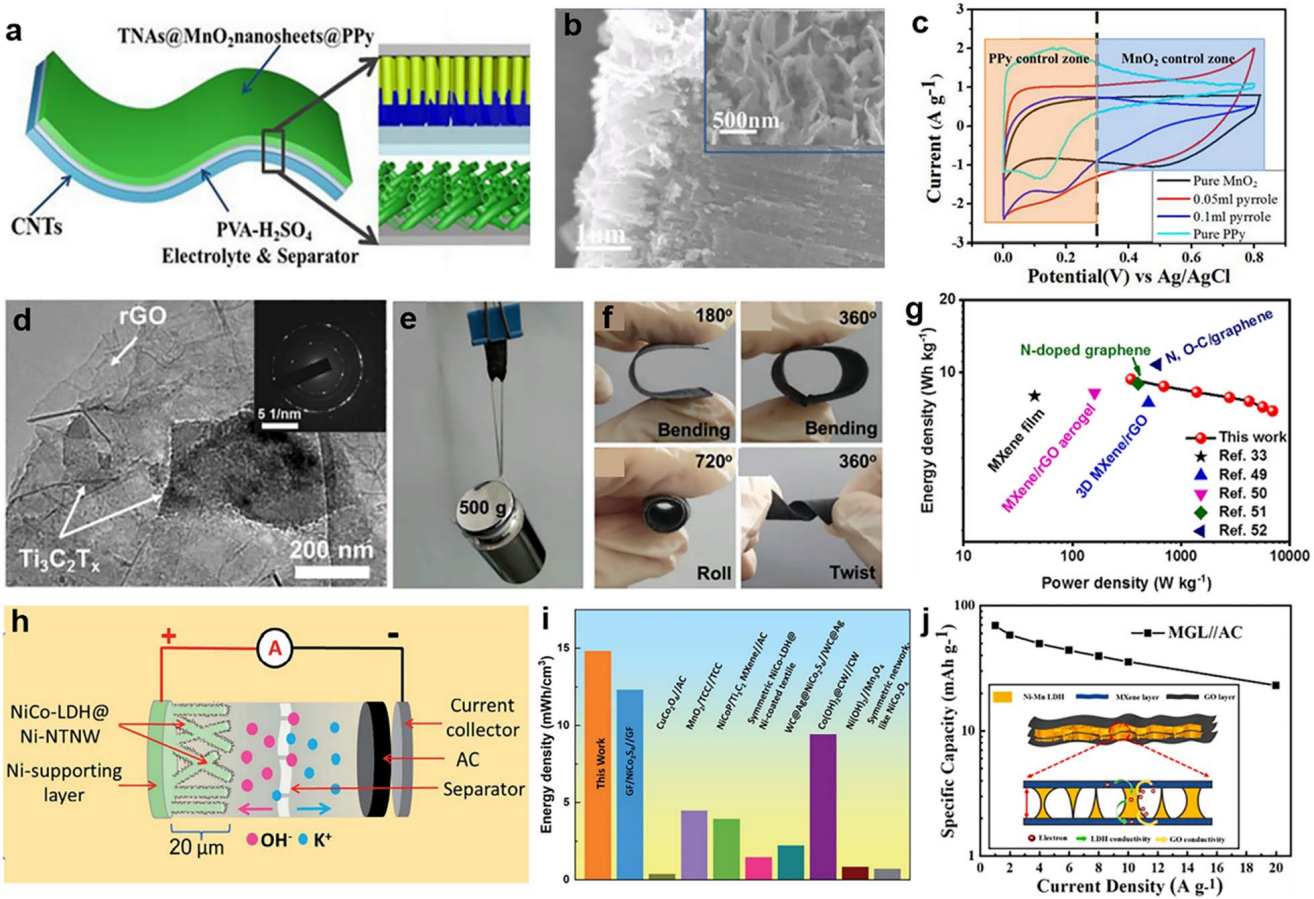
2D nanomaterials for energy. **a** Scheme of TNAs@MnO_2_ nanosheets@PPy.** b** The morphology of TMP. **c** The CV curves of designed TMP. **d** Morphology of MXene/rGO/carbon hybrid electrode. **e** Mechanical performance of composite films. **f** The flexibility of composite films. **g** Performance of prepared supercapacitors. **h** Scheme of supercapacitors. **i** The energy storage performance of reported supercapacitors. **j** Specific capacity of MXene/GO/LDH nanocomposite at various current densities (insert: Conducting mechanism). **a**–**c** Reproduced with permission [86]. Copyright 2017, Elsevier B.V. **d**–**g** Reproduced with permission [87]. Copyright 2021, Elsevier B.V. **h**, **i** Reproduced with permission [88]. Copyright 2021, The Royal Society of Chemistry. **j** Reproduced with permission [89]. Copyright 2021, Elsevier Ltd

Zhu et al. developed a unique 3D-nanostructured electrode comprising TiO_2_ nanotube array, MnO_2_ nanosheet, and polypyrrole, capable of functioning in acidic conditions (Fig. 7a–c). The constructed electrode was used to assemble an asymmetric supercapacitor, showcasing impressive performance metrics: a high energy density, a robust power density, and excellent cycling stability, retaining over 80% of its capacity after 20,000 cycles. The impressive cycling stability was attributed to the protective role of the polypyrrole shell surrounding the internal MnO_2_. This shell acted as a barrier, restricting the dissolution of MnO_2_ during the discharge process [86].

According to reports, 2D MXene materials are excellent substrates for creating supercapacitors. This is because they contain functional groups such as -F, -OH, or -O, which enhance the interaction of positively charged monomers and enable the polymerization of conductive polymers [90]. Zhang et al. introduced hybrid electrodes for wearable supercapacitors using Ti_3_C_2_Tx MXene. They transformed 2D MXene sheets into a sturdy, interconnected cellular structure, and added rGO as a conductive binder through strong π-π stacking between the sheets. This hybrid MXene foam demonstrated excellent compressibility and electrochemical performance, making it suitable for wearable applications (Fig. 7d–g) [87]. To enhance the performance of SCs, researchers blend conductive polymers with 2D MXene materials to make flexible SCs, which gained significant interest because it improve the charge transfer, leading to enhanced energy densities. For instance, Jian et al. developed a 3D MXene/polypyrrole composite electrodes using an in situ chemical polymerization. The created 3D structured composite promoted electron and ionic transport, resulting in symmetric SCs with a high specific capacitance. Additionally, these SCs exhibited remarkable reliability and maintained approximately 86.4% retention for 5000 cycles cycling. This development offers the potential for preparing high-performance composite devices based on 2D MXene [91].

Layered double hydroxide (LDH) is a class of 2D materials with great potential for preparing high-performance flexible SCs [92]. To meet this energy requirement, Liang et al. introduced a high-performance flexible supercapacitor designed for wearable storage devices. This supercapacitor features Co-Ni LDH nanosheets as the positive electrode. The deposited Co-Ni LDH nanomaterial on the pregnant woman cloth, given full play to the porous and flexible characteristics of 2D materials. The prepared supercapacitors exhibited impressive attributes, including remarkable power density and energy density, excellent flexibility, and stability. These characteristics make them suitable for serving as energy storage devices [93]. Amin et al. created hierarchical morphology of Ni/NiCo-LDH nanotube networks, forming a self-supporting electrode for SCs. The resulting 3D core–shell structure combined the benefits of the Ni network core, including outstanding conductivity and rapid electron transfer, with the advantages of 2D NiCo-LDH nanosheets, such as increased reactive sites. The results demonstrated that the structure can endow SCs with high volumetric energy density (Fig. 7h, i) [88].

To leverage the diverse characteristics of 2D nanomaterials, researchers prepared sandwich-structured 2D MXene/GO/LDH composites by integrating 2D nanomaterials. Chen et al. prepared a sandwich-like MXene/GO/LDH nano matrix for high performance supercapacitor, demonstrating distinctive structure that delivers exceptional performance (Fig. 7j). Owing to its heterogeneous structure, avoiding the issues of the stacking of MXene. Additionally, it prevents the collapse of LDH morphology, significantly enhancing LDH's specific capacity. Meanwhile, GO serves as a thin layer, coating the surface of nanocomposite supercapacitor with a precise structure, expediting charge transfer, and increasing the electron density of the materials. As a result, he combined effect of various composites enhances the active sites and boosts the electrode’s electroactivity in redox reactions. This improvement enables the asymmetric supercapacitor to achieve a high specific energy while maintaining a remarkable capacity retention [89].

**Fig. 8:**
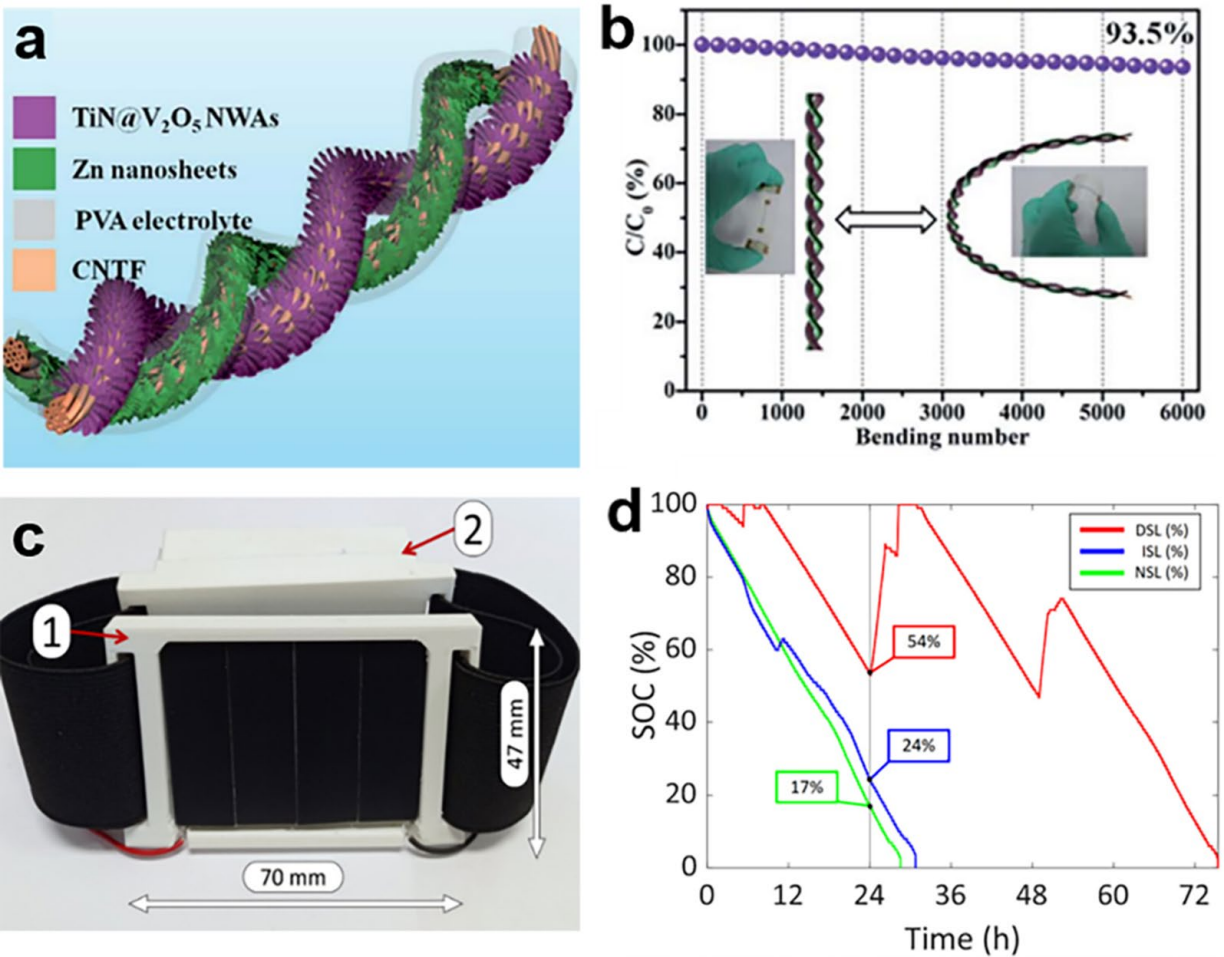
Emerging wearable electronic devices. **a** Schematic illustration of the structure of zinc-ion batteries. **b** The CV of the zinc-ion batteries. **c** Wearable device prototype including solar cells module housing and flexible battery housing. **d** State-of-Charge of the wearable device. **a**, **b** Reproduced with permission [94]. Copyright 2019, Royal Society of Chemistry. **c**, **d** Reproduced with permission [95] Copyright 2010, the authors

The rise of wearable electronics has led to a growing need for flexible energy storage devices [96, 97]. Flexible LIBs offer higher energy density, but their inherent safety concerns and potential environmental issues have hindered their wide applications; Flexible supercapacitors have the advantage of outstanding power density, yet their limited energy density and short discharge duration hinder their practical use. Zinc-ion batteries (ZIBs) have attracted increasing attention in wearable electronics, due to their noteworthy features of high theoretical capacity and affordability [98]. Among them, the aqueous Zn/MnO_2_ battery with ZnSO_4_/MnSO_4_ as the electrolyte has the advantages of wide electrochemical window, high specific capacity, low cost, environmental protection and simple fabrication, and is considered to be one of the most potential candidate in flexible ZIBs [99]. For instance, Li et al. fabricated a hierarchical core–shell structure composed of well-aligned nanowire arrays. This structure was achieved by in-situ growing TiN and V_2_O_5_ on the surface of plasma-treated carbon nanotube fibers through a hydrothermal method (Fig. 8a). Benefiting from the synergistic effect of TiN's inherent high conductivity, good interfacial buffering effect, and the high capacity of sheet-like V_2_O_5_, the aqueous ZIBs exhibited outstanding performance, demonstrating excellent flexibility and integration capabilities. Additionally, ZIBs displayed good cycle stability with a capacity retention of 90.6% after 3500 cycles (Fig. 8b) [94].

Electrochemical catalysis can be viewed as the process of converting electrical energy into chemical energy [100]. Similarly, a solar cell transforms light energy into electrical energy by utilizing the photoelectric or photochemical effect [101]. Carbon nanomaterials demonstrate remarkable electrical conductivity and strong light absorption capabilities, resulting in effective photodegradation and photocatalysis process. These qualities also render them ideal for use in solar cells (Fig. 8c, d) [95]. Researchers have developed solar cells according to Schottky junctions within graphene nanosheets and Si. The graphene nanosheet film serves a dual purpose: it acts as a transparent electrode with exceptional light transmission and functions as a Schottky junction layer for separating electron–hole pairs and facilitating hole transport. As a result, the photogenerated carriers are separated, causing holes and electrons to diffuse to both ends of the graphene and silicon, respectively [102, 103].

### 2D nanocomposite flexible fabric materials for wearable electronics

Throughout the ages, clothes have always been necessary to wear, which can be beautiful embroidered silk, ragged clothes, night walks in brocade clothes, comfortable in warmth and coldness. A piece of clothing, from the basic function of sheltering from the wind and cold, to the obsession with beautiful things, the perception of success and fame, and finally evolves into people's ultimate demand for health. With the popularization of 5G technology, electronic fabrics are fundamentally changing daily lives by interacting with the human body and the environment [104–106]. Clothes, as a new type of carrier, can continuously obtain human physiological information, bringing disease prevention and health protection into the era of personalized precision medicine [107]. One of the keys to the advancement of flexible wearable devices like e-textiles is the stable operation of the power supply. Wearable fabrics range from professional sportswear to everyday apparel [108]. The difficulty lies in creating electronic clothing that is as comfortable, soft and washable as other traditional clothing. Interconnects and electronics must be robust and unobtrusive. This requires: insulated, robust, and waterproof reliable terminals; flexible, clothing-based antenna and transceiver solutions; insulated wires that are elastically conductive; small, dryable batteries; and materials that are wrinkle- and curl-resistant [109].

**Fig. 9:**
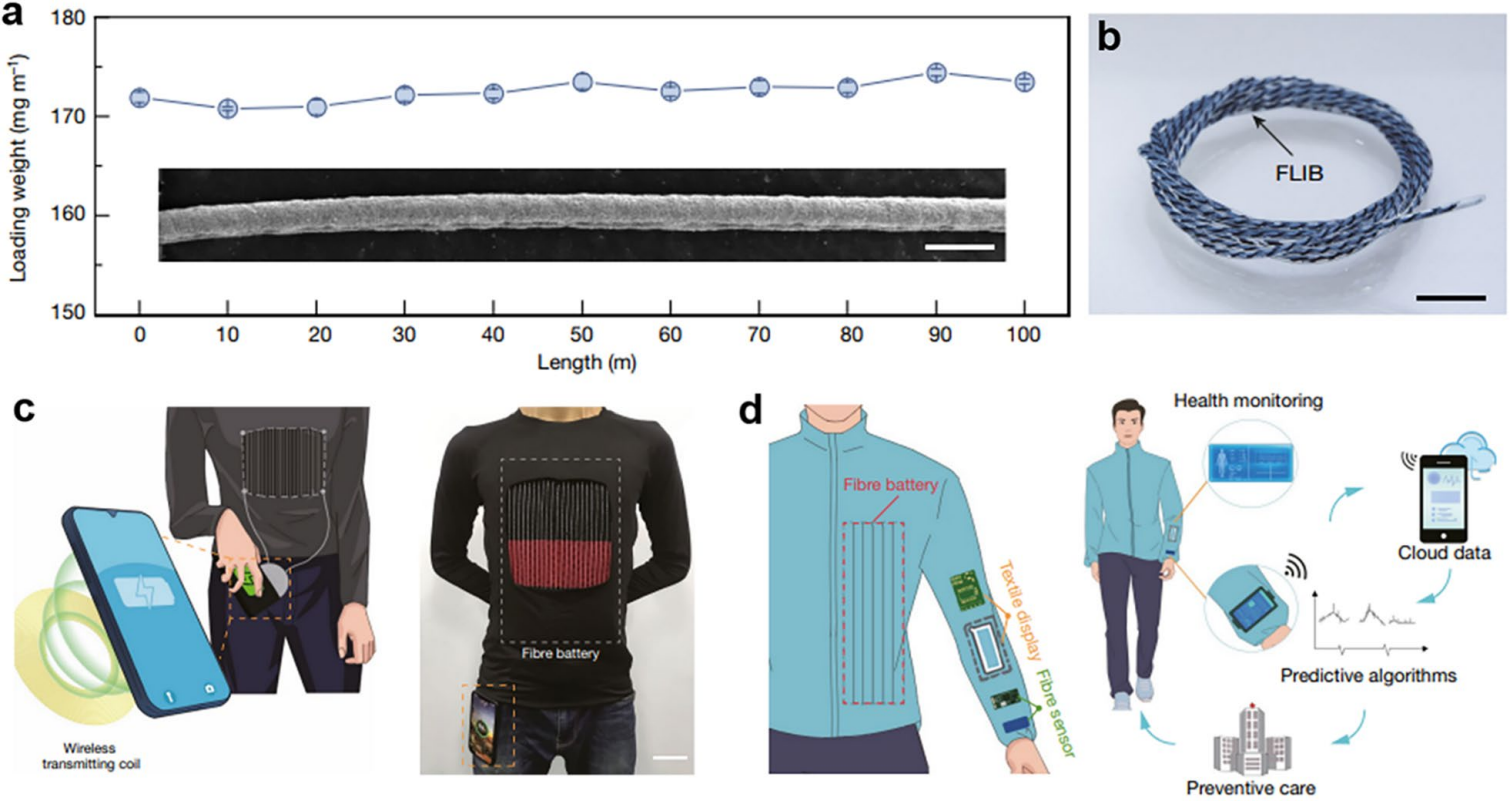
Scalable production of 2D nanocomposite flexible fabric materials. **a** The maintained loading weight of fibre electrode as the electrode length increasing. **b** Magnified photograph of lithium-ion fibre batteries with a length of 1 m. **c** Illustration showing of lithium-ion fibre batteries textile for the applications for charging a mobile phone. **d** The applications of wearable textile for powering and displaying. Reproduced with permission [110] Copyright 2021, Springer Nature

Over the past few decades, significant strides have been taken in the fields of flexible SCs and LIBs to cater to the demands of wearable bioelectronic devices [111]. In terms of industrial scale-up, Peng's research team has successively carried out methodological research on continuous battery assembly and packaging, and finally realized the continuous and stable preparation of high-performance fiber LIBs (Fig. 9) [110]. The capacity of the fiber LIB increases linearly with the length. Considering its total mass, a 1 m-long fiber LIB boasts an energy density exceeding 80 Wh kg^−1^, enabling it to power commercial wearable devices like heart rate monitors for over 2 days continuously. At the same time, the fiber battery fabric can work normally under harsh conditions such as bending, dynamic deformation, high and low temperature (− 20–60 °C), puncture and washing, showing good application potential. The research team further obtained a high-performance and high-safety large-area battery fabric through the textile method, and carried out some functional demonstrations in real application scenarios, allowing for safe and stable wireless charging of a smartphone kept in the wearer’s pocket. The whole process lasted for 40 min, and no obvious heating phenomenon occurred, showing good safety. In addition, the research team integrated the fiber LIB and fiber sensor with the display fabric.

During exercise, the fiber sensor measures sodium and calcium ion levels in sweat. It sends this data to a chip, which then transmits the signal to the screen. This provides the possibility for the application of smart medical care in the future. The challenge in the electronic wearables market is to create devices that can provide useful data to help improve our lives. Whether worn on the wrist, head or feet, wearables must be stylish, durable and easily rechargeable [109].

## Conclusions and outlook

In this article, we have discussed recent advancements in sustainable wearable energy devices using 2D nanomaterials. It is evident that research activities and published work around the world have seen a rapid increase. Numerous promising applications in health, medical care, energy, smart city, sports, and other fields have been explored and demonstrated. In summary, 2D nanomaterials exhibit ideal traits for flexible sensor components in wearable devices, including a substantial specific surface area, high electrical conductivity, and exceptional mechanical properties. The article reviews the applications of various 2D nanomaterials in flexible wearable devices, detailing their sensing properties and mechanisms. Meanwhile, this article covers several main applications of 2D nanomaterials based sustainable wearable energy devices, such as medical health, motion detection and electronic skin, and energy and its composite material for flexible fabrics. The results show that MXene and graphene, among 2D nanomaterials, possess excellent electrical conductivity, flexibility, and mechanical stability, and their morphology and structure can be adjusted, making 2D nanomaterials great promise for the advancement of flexible sensors. The vigorous development and practical application of next-generation flexible electronics based on 2D nanomaterials are still very promising. The development of fiber-based smart textiles is very important for the realization of mass manufactured and widely used wearable electronic devices in the future, however, there is still a long way to go to. Despite notable advancements in creating flexible piezoresistive nanomaterials, there are still unresolved issues in their application for wearable energy devices.

- Challenges in 2D material production for flexible wearable devices:

The primary hurdle in the realm of 2D materials revolves around the economical and uniform production of defect-free 2D thin layers. It is difficult to synthesize 2D sensing materials in a tunable manner. For example, preparing 2D nanosheets with a specific size, surface functional group modification, and arbitrary spatial distribution is difficult. Existing methods, while effective, are either time-intensive or costly, lacking scalability for large-scale preparations of flexible wearable energy devices. A critical need persists for technologies enabling the cost-efficient, uniform production of these layers, vital for the advancement of flexible energy solutions.

b) Developing high-performance 2D nanomaterials for flexible piezoresistive sensors:

Among the plethora of 2D nanomaterials, only a handful find application in flexible piezoresistive sensors. It is crucial to concentrate on creating high-performance 2D nanomaterials designed specifically for these sensors. Enhancing the properties of these materials is essential, ensuring their seamless integration into flexible piezoresistive sensors for optimal performance, thereby expanding the scope of their applications.

c) Conformity and accuracy in soft body wearables:

The human body, inherently soft, contrasts starkly with current rigid wearable sensors, leading to mechanical mismatches in Young’s moduli. This rigidity impedes the establishment of conformal contact, causing dislocation during body movements and compromising data accuracy. Maintaining precision in biometric information is paramount; inaccuracies could render the data unusable for health assessments. Overcoming this challenge requires the development of flexible sensors that can seamlessly adhere to the body's contours, ensuring accurate and reliable data acquisition.

d) Expanding wearable design and comfort:

Contemporary “wearables” are confined to conventional gadgets like wristbands, watches, glasses, and earrings. This limited design spectrum imposes constraints, leading to discomfort and awkward wearability. These rigid devices not only limit sensor positions but also curtail the duration for which readings can be obtained comfortably. Innovations in wearable design, including the exploration of novel form factors and materials, are imperative. Embracing flexible and ergonomic designs will not only enhance user comfort but also augment the usability and acceptance of wearable technologies in diverse contexts.

## Data Availability

Not applicable.

## References

[1] Mokhtari F, Cheng Z, Raad R, Xi J, Foroughi J (2021). Piezofibers to smart textiles: a review on recent advances and future outlook for wearable technology. J Mater Chem A.

[2] Kim H, Pyun K, Lee M-T, Lee HB, Ko SH (2022). Recent advances in sustainable wearable energy devices with nanoscale materials and macroscale structures. Adv Funct Mater.

[3] Katrin A, Thomas L (2021). Brain research challenges supercomputing. Science.

[4] Wang J, Dong J (2020). Optical waveguides and integrated optical devices for medical diagnosis, health monitoring and light therapies. Sensors.

[5] Kim J, Campbell AS, de Avila BE, Wang J (2019). Wearable biosensors for healthcare monitoring. Nat Biotechnol.

[6] Zhang Y, Xu Z, Yuan Y, Liu C, Zhang M, Zhang L, Wan P (2023). Flexible antiswelling photothermal-therapy MXene hydrogel-based epidermal sensor for intelligent human-machine interfacing. Adv Funct Mater.

[7] Xia S, Wang M, Gao G (2022). Preparation and application of graphene-based wearable sensors. Nano Res.

[8] Yin R, Wang D, Zhao S, Lou Z, Shen G (2020). Wearable sensors-enabled human-machine interaction systems: from design to application. Adv Funct Mater.

[9] Aragay G, Pino F, Merkoci A (2012). Nanomaterials for sensing and destroying pesticides. Chem Rev.

[10] Tiwari JN, Tiwari RN, Kim KS (2012). Zero-dimensional, one-dimensional, two-dimensional and three-dimensional nanostructured materials for advanced electrochemical energy devices. Prog Mater Sci.

[11] Kaul AB (2014). Two-dimensional layered materials: structure, properties, and prospects for device applications. J Mater Res.

[12] Chimene D, Alge DL, Gaharwar AK (2015). Two-dimensional nanomaterials for biomedical applications: emerging trends and future prospects. Adv Mater.

[13] Osada M, Sasaki T (2012). Two-dimensional dielectric nanosheets: novel nanoelectronics from nanocrystal building blocks. Adv Mater.

[14] Kannan PK, Late DJ, Morgan H, Rout CS (2015). Recent developments in 2D layered inorganic nanomaterials for sensing. Nanoscale.

[15] Orts V, Chan KC, Caironi M, Athanassiou A, Kinloch IA, Bissett M, Cataldi P (2022). Electrically conductive 2D material coatings for flexible and stretchable electronics: a comparative review of graphenes and MXenes. Adv Funct Mater.

[16] Ismail SNA, Nayan NA, Mohammad Haniff MAS, Jaafar R, May Z (2023). Wearable two-dimensional nanomaterial-based flexible sensors for blood pressure monitoring: a review. Nanomaterials.

[17] Novoselov KS, Geim AK, Morozov SV, Jiang D, Zhang Y, Dubonos SV, Grigorieva IV, Firsov AA (2004). Electric field effect in atomically thin carbon films. Science.

[18] Glavin NR, Rao R, Varshney V, Bianco E, Apte A, Roy A, Ringe E, Ajayan PM (2020). Emerging applications of elemental 2D materials. Adv Mater.

[19] Liu Y, Huang Y, Duan X (2019). Van der Waals integration before and beyond two-dimensional materials. Nature.

[20] Sempionatto JR, Lasalde-Ramírez JA, Mahato K, Wang J, Gao W (2022). Wearable chemical sensors for biomarker discovery in the omics era. Nat Rev Chem.

[21] Xu W, Hu S, Zhao Y, Zhai W, Chen Y, Zheng G, Dai K, Liu C, Shen C (2021). Nacre-inspired tunable strain sensor with synergistic interfacial interaction for sign language interpretation. Nano Energy.

[22] Farjadian F, Abbaspour S, Sadatlu MAA, Mirkiani S, Ghasemi A, Hoseini-Ghahfarokhi M, Mozaffari N, Karimi M, Hamblin MR (2020). Recent developments in graphene and graphene oxide: properties, synthesis, and modifications: a review. ChemistrySelect.

[23] Zhou Y, Dai D, Gao Y, Zhang Z, Sun N, Tan H, Cai X, Cai J (2021). Recent advances in graphene electronic skin and its future prospects. ChemNanoMat.

[24] Wan S, Bi H, Zhou Y, Xie X, Su S, Yin K, Sun L (2017). Graphene oxide as high-performance dielectric materials for capacitive pressure sensors. Carbon.

[25] Pang Y, Jian J, Tu T, Yang Z, Ling J, Li Y, Wang X, Qiao Y, Tian H, Yang Y, Ren TL (2018). Wearable humidity sensor based on porous graphene network for respiration monitoring. Biosens Bioelectron.

[26] Wei Y, Li X, Wang Y, Hirtz T, Guo Z, Qiao Y, Cui T, Tian H, Yang Y, Ren TL (2021). Graphene-based multifunctional textile for sensing and actuating. ACS Nano.

[27] Huang T, He P, Wang R, Yang S, Sun J, Xie X, Ding G (2019). Porous fibers composed of polymer nanoball decorated graphene for wearable and highly sensitive strain sensors. Adv Funct Mater.

[28] Chen X, Park YJ, Kang M, Kang SK, Koo J, Shinde SM, Shin J, Jeon S, Park G, Yan Y, MacEwan MR, Ray WZ, Lee KM, Rogers JA, Ahn JH (2018). CVD-grown monolayer MoS2 in bioabsorbable electronics and biosensors. Nat Commun.

[29] Lee I, Kang WT, Shin YS, Kim YR, Won UY, Kim K, Duong DL, Lee K, Heo J, Lee YH, Yu WJ (2019). Ultrahigh gauge factor in graphene/MoS(2) heterojunction field effect transistor with variable schottky barrier. ACS Nano.

[30] Wang H, Yuan H, Sae Hong S, Li Y, Cui Y (2015). Physical and chemical tuning of two-dimensional transition metal dichalcogenides. Chem Soc Rev.

[31] Chowdhury T, Sadler EC, Kempa TJ (2020). Progress and prospects in transition-metal dichalcogenide research beyond 2D. Chem Rev.

[32] Chhowalla M, Shin HS, Eda G, Li LJ, Loh KP, Zhang H (2013). The chemistry of two-dimensional layered transition metal dichalcogenide nanosheets. Nat Chem.

[33] Tajik S, Dourandish Z, Garkani Nejad F, Beitollahi H, Jahani PM, Di Bartolomeo A (2022). Transition metal dichalcogenides: synthesis and use in the development of electrochemical sensors and biosensors. Biosens Bioelectron.

[34] Park YJ, Sharma BK, Shinde SM, Kim MS, Jang B, Kim JH, Ahn JH (2019). All MoS(2)-based large area, skin-attachable active-matrix tactile sensor. ACS Nano.

[35] Park S, Lee A, Choi KH, Hyeong SK, Bae S, Hong JM, Kim TW, Hong BH, Lee SK (2020). Layer-selective synthesis of MoS(2) and WS(2) structures under ambient conditions for customized electronics. ACS Nano.

[36] Murali G, Reddy Modigunta JK, Park YH, Lee JH, Rawal J, Lee SY, In I, Park SJ (2022). A review on MXene synthesis, stability, and photocatalytic applications. ACS Nano.

[37] Liu J, Zhang HB, Sun R, Liu Y, Liu Z, Zhou A, Yu ZZ (2017). Hydrophobic, flexible, and lightweight MXene foams for high-performance electromagnetic-interference shielding. Adv Mater.

[38] Zhao Z, Xia K, Hou Y, Zhang Q, Ye Z, Lu J (2021). Designing flexible, smart and self-sustainable supercapacitors for portable/wearable electronics: from conductive polymers. Chem Soc Rev.

[39] Zhang K, Sun J, Song J, Gao C, Wang Z, Song C, Wu Y, Liu Y (2020). Self-healing Ti(3)C(2) MXene/PDMS supramolecular elastomers based on small biomolecules modification for wearable sensors. ACS Appl Mater Interfaces.

[40] Ren A, Zou J, Lai H, Huang Y, Yuan L, Xu H, Shen K, Wang H, Wei S, Wang Y, Hao X, Zhang J, Zhao D, Wu J, Wang Z (2020). Direct laser-patterned MXene–perovskite image sensor arrays for visible-near infrared photodetection. Mater Horiz.

[41] Chao M, Wang Y, Ma D, Wu X, Zhang W, Zhang L, Wan P (2020). Wearable MXene nanocomposites-based strain sensor with tile-like stacked hierarchical microstructure for broad-range ultrasensitive sensing. Nano Energy.

[42] Li Y, Zhang X (2021). Electrically conductive, optically responsive, and highly orientated Ti3C2Tx MXene aerogel fibers. Adv Funct Mater.

[43] de Souza FAL, Sivaraman G, Hertkorn J, Amorim RG, Fyta M, Scopel WL (2019). Hybrid 2D nanodevices (graphene/h-BN): selecting NOx gas through the device interface. J Mater Chem A.

[44] Liu G, Tang Y, Soomro AM, Shen P, Lu S, Cai Y, Wang H, Yang Q, Chen H, Shi Y, Lin C, Xu F, Xu F, Wu Z, Chen X, Cai D, Kang J (2023). Vertically aligned ZnO nanoarray directly orientated on Cu paper by h-BN monolayer for flexible and transparent piezoelectric nanogenerator. Nano Energy.

[45] Wu Y, Yuan W, Xu M, Bai S, Chen Y, Tang Z, Wang C, Yang Y, Zhang X, Yuan Y, Chen M, Zhang X, Liu B, Jiang L (2021). Two-dimensional black phosphorus: properties, fabrication and application for flexible supercapacitors. Chem Eng J.

[46] Eswaraiah V, Zeng Q, Long Y, Liu Z (2016). Black phosphorus nanosheets: synthesis, characterization and applications. Small.

[47] Li Q, Wu JT, Liu Y, Qi XM, Jin HG, Yang C, Liu J, Li GL, He QG (2021). Recent advances in black phosphorus-based electrochemical sensors: a review. Anal Chim Acta.

[48] Huo Z, Wei Y, Wang Y, Wang ZL, Sun Q (2022). Integrated self-powered sensors based on 2D material devices. Adv Funct Mater.

[49] Peng Y, Yang N, Xu Q, Dai Y, Wang Z (2021). Recent advances in flexible tactile sensors for intelligent systems. Sensors.

[50] Stassi S, Cauda V, Canavese G, Pirri CF (2014). Flexible tactile sensing based on piezoresistive composites: a review. Sensors.

[51] Lu X, Yang J, Qi L, Bao W, Zhao L, Chen R (2019). High sensitivity flexible electronic skin based on graphene film. Sensors.

[52] Sengupta D, Pei Y, Kottapalli AGP (2019). Ultralightweight and 3D squeezable graphene-polydimethylsiloxane composite foams as piezoresistive sensors. ACS Appl Mater Interfaces.

[53] Wang S, Chen G, Niu S, Chen K, Gan T, Wang Z, Wang H, Du P, Leung CW, Qu S (2019). Magnetic-assisted transparent and flexible percolative composite for highly sensitive piezoresistive sensor via hot embossing technology. ACS Appl Mater Interfaces.

[54] Wu Z, Wei J, Dong R, Chen H (2019). Epoxy composites with reduced graphene oxide-cellulose nanofiber hybrid filler and their application in concrete strain and crack monitoring. Sensors.

[55] Yu X, Zhang W, Zhang P, Su Z (2017). Fabrication technologies and sensing applications of graphene-based composite films: advances and challenges. Biosens Bioelectron.

[56] Yang T, Xiang H, Qin C, Liu Y, Zhao X, Liu H, Li H, Ouzounian M, Hong G, Chen H, Dong Q, Hu TS, Liu S (2019). Highly sensitive 1T-MoS2 pressure sensor with wide linearity based on hierarchical microstructures of leaf vein as spacer. Adv Electron Mater.

[57] Liu W, Cheng Y, Liu N, Yue Y, Lei D, Su T, Zhu M, Zhang Z, Zeng W, Guo H, Gao Y (2021). Bionic MXene actuator with multiresponsive modes. Chem Eng J.

[58] Ma Y, Cheng Y, Wang J, Fu S, Zhou M, Yang Y, Li B, Zhang X, Nan CW (2022). Flexible and highly-sensitive pressure sensor based on controllably oxidized MXene. InfoMat.

[59] Wang Y, Yue Y, Cheng F, Cheng Y, Ge B, Liu N, Gao Y (2022). Ti(3)C(2)T(x) MXene-based flexible piezoresistive physical sensors. ACS Nano.

[60] Ma C, Ma MG, Si C, Ji XX, Wan P (2021). Flexible MXene-based composites for wearable devices. Adv Funct Mater.

[61] Minev IR (2015). Electronic dura mater for long-term multimodal neural interfaces. Science.

[62] Choi S, Lee H, Ghaffari R, Hyeon T, Kim DH (2016). Recent advances in flexible and stretchable bio-electronic devices integrated with nanomaterials. Adv Mater.

[63] Kabiri Ameri S, Ho R, Jang H, Tao L, Wang Y, Wang L, Schnyer DM, Akinwande D, Lu N (2017). Graphene electronic tattoo sensors. ACS Nano.

[64] Kuzum D, Takano H, Shim E, Reed JC, Juul H, Richardson AG, de Vries J, Bink H, Dichter MA, Lucas TH, Coulter DA, Cubukcu E, Litt B (2014). Transparent and flexible low noise graphene electrodes for simultaneous electrophysiology and neuroimaging. Nat Commun.

[65] Zhao J, Li N, Yu H, Wei Z, Liao M, Chen P, Wang S, Shi D, Sun Q, Zhang G (2017). Highly sensitive MoS(2) humidity sensors array for noncontact sensation. Adv Mater.

[66] Zhang Z, Wang W, Jiang Y, Wang YX, Wu Y, Lai JC, Niu S, Xu C, Shih CC, Wang C, Yan H, Galuska L, Prine N, Wu HC, Zhong D, Chen G, Matsuhisa N, Zheng Y, Yu Z, Wang Y, Dauskardt R, Gu X, Tok JB, Bao Z (2022). High-brightness all-polymer stretchable LED with charge-trapping dilution. Nature.

[67] Choi MK (2015). Thermally controlled, patterned graphene transfer printing for transparent and wearable electronic/optoelectronic system. Adv Func Mater.

[68] Lee H (2015). An endoscope with integrated transparent bioelectronics and theranostic nanoparticles for colon cancer treatment. Nat Commun.

[69] Zang J, Ryu S, Pugno N, Wang Q, Tu Q, Buehler MJ, Zhao X (2013). Multifunctionality and control of the crumpling and unfolding of large-area graphene. Nat Mater.

[70] Park M, Park YJ, Chen X, Park YK, Kim MS, Ahn JH (2016). MoS2 -based tactile sensor for electronic skin applications. Adv Mater.

[71] Kim SJ, Cho HR, Cho KW, Qiao S, Rhim JS, Soh M, Kim T, Choi MK, Choi C, Park I, Hwang NS, Hyeon T, Choi SH, Lu N, Kim DH (2015). Multifunctional cell-culture platform for aligned cell sheet monitoring, transfer printing, and therapy. ACS Nano.

[72] Yang Q (2021). Photocurable bioresorbable adhesives as functional interfaces between flexible bioelectronic devices and soft biological tissues. Nat Mater.

[73] Alex A, Carmel TC, Yasser K, Alana M-B, Naoji M, Robyn F, Rohan S, William H, Parag M, Sanjiv SG, Bao Z (2022). A flexible electronic strain sensor for the real-time monitoring of tumor regression. Sci Adv.

[74] Hou C, Tai G, Liu Y, Liu R, Liang X, Wu Z, Wu Z (2022). Borophene pressure sensing for electronic skin and human-machine interface. Nano Energy.

[75] Yun YJ, Ju J, Lee JH, Moon S-H, Park S-J, Kim YH, Hong WG, Ha DH, Jang H, Lee GH, Chung H-M, Choi J, Nam SW, Lee S-H, Jun Y (2017). Highly elastic graphene-based electronics toward electronic skin. Adv Funct Mater.

[76] Zhao J, Wei Z, Li Z, Yu J, Tang J, Zhang G, Wang Z (2021). Skin-inspired high-performance active-matrix circuitry for multimodal user-interaction. Adv Funct Mater.

[77] Ekaterina P, Francesco B, Feng X, Cui Y, Yury G (2019). Energy storage: the future enabled by nanomaterials. Science.

[78] Zhang Q, Uchaker E, Candelaria SL, Cao G (2013). Nanomaterials for energy conversion and storage. Chem Soc Rev.

[79] Zhang H, Chhowalla M, Liu Z (2018). 2D nanomaterials: graphene and transition metal dichalcogenides. Chem Soc Rev.

[80] Hu Y, Sun X (2014). Flexible rechargeable lithium ion batteries: advances and challenges in materials and process technologies. J Mater Chem A.

[81] Li M, Lu J, Chen Z, Amine K (2018). 30 years of lithium-ion batteries. Adv Mater.

[82] Chen D, Lou Z, Jiang K, Shen G (2018). Device configurations and future prospects of flexible/stretchable lithium-ion batteries. Adv Funct Mater.

[83] Wang J, Wang L, Feng J, Tang C, Sun X, Peng H (2021). Long-term in vivo monitoring of chemicals with fiber sensors. Adv Fiber Mater.

[84] Zheng S, Ma J, Wu Z-S, Zhou F, He Y-B, Kang F, Cheng H-M, Bao X (2018). All-solid-state flexible planar lithium ion micro-capacitors. Energy Environ Sci.

[85] Lin X, Li X, Yang N, Li X, Yao J, Zhang W, Yan R, Xu J, Komarneni S (2023). Design and construction of 1D/2D/3D fabric-based wearable micro-supercapacitors. J Power Sources.

[86] Zhu Q, Liu K, Zhou J, Hu H, Chen W, Yu Y (2017). Design of a unique 3D-nanostructure to make MnO2 work as supercapacitor material in acid environment. Chem Eng J.

[87] Zhang J, Jiang D, Liao L, Cui L, Zheng R, Liu J (2022). Ti3C2T MXene based hybrid electrodes for wearable supercapacitors with varied deformation capabilities. Chem Eng J.

[88] Amin KM, Krois K, Muench F, Etzold BJM, Ensinger W (2022). Hierarchical pipe cactus-like Ni/NiCo-LDH core–shell nanotube networks as a self-supported battery-type electrode for supercapacitors with high volumetric energy density. J Mater Chem A.

[89] Chen M, Chen J, Tan X, Yang W, Zou H, Chen S (2021). Facile self-assembly of sandwich-like MXene/graphene oxide/nickel–manganese layered double hydroxide nanocomposite for high performance supercapacitor. J Energy Storage.

[90] Gao B, Li X, Ding K, Huang C, Li Q, Chu PK, Huo K (2019). Recent progress in nanostructured transition metal nitrides for advanced electrochemical energy storage. J Mater Chem A.

[91] Jian X, He M, Chen L, Zhang M-m, Li R, Gao L-j, Fu F, Liang Z-h (2019). Three-dimensional carambola-like MXene/polypyrrole composite produced by one-step co-electrodeposition method for electrochemical energy storage. Electrochim Acta.

[92] Li X, Du D, Zhang Y, Xing W, Xue Q, Yan Z (2017). Layered double hydroxides toward high-performance supercapacitors. J Mater Chem A.

[93] Liang X, Long G, Fu C, Pang M, Xi Y, Li J, Han W, Wei G, Ji Y (2018). High performance all-solid-state flexible supercapacitor for wearable storage device application. Chem Eng J.

[94] Li Q, Zhang Q, Liu C, Zhou Z, Li C, He B, Man P, Wang X, Yao Y (2019). Anchoring V2O5 nanosheets on hierarchical titanium nitride nanowire arrays to form core–shell heterostructures as a superior cathode for high-performance wearable aqueous rechargeable zinc-ion batteries. J Mater Chem A.

[95] Borchers A, Pieler T (2010). Programming pluripotent precursor cells derived from Xenopus embryos to generate specific tissues and organs. Genes.

[96] Xiang F, Cheng F, Sun Y, Yang X, Lu W, Amal R, Dai L (2021). Recent advances in flexible batteries: from materials to applications. Nano Res.

[97] Huang S, Liu Y, Zhao Y, Ren Z, Guo CF (2018). Flexible electronics: stretchable electrodes and their future. Adv Funct Mater.

[98] Yu P, Zeng Y, Zhang H, Yu M, Tong Y, Lu X (2019). Flexible Zn-ion batteries: recent progresses and challenges. Small.

[99] Dong H, Li J, Guo J, Lai F, Zhao F, Jiao Y, Brett DJL, Liu T, He G, Parkin IP (2021). Insights on flexible zinc-ion batteries from lab research to commercialization. Adv Mater.

[100] Gao D, Li H, Wei P, Wang Y, Wang G, Bao X (2022). Electrochemical synthesis of catalytic materials for energy catalysis. Chinese J Catal.

[101] Hashemi SA, Ramakrishna S, Aberle AG (2020). Recent progress in flexible–wearable solar cells for self-powered electronic devices. Energy Environ Sci.

[102] Kim H, Ahn J-H (2017). Graphene for flexible and wearable device applications. Carbon.

[103] Das T, Sharma BK, Katiyar AK, Ahn J-H (2018). Graphene-based flexible and wearable electronics. J Semicond.

[104] Chen G, Xiao X, Zhao X, Tat T, Bick M, Chen J (2022). Electronic textiles for wearable point-of-care systems. Chem Rev.

[105] Du K, Lin R, Yin L, Ho JS, Wang J, Lim CT (2022). Electronic textiles for energy, sensing, and communication. iScience.

[106] Shi HH, Pan Y, Xu L, Feng X, Wang W, Potluri P, Hu L, Hasan T, Huang YYS (2023). Sustainable electronic textiles towards scalable commercialization. Nat Mater.

[107] Ling Y, An T, Yap LW, Zhu B, Gong S, Cheng W (2020). Disruptive, soft, wearable sensors. Adv Mater.

[108] Xiao X (2021). An ultrathin rechargeable solid-state zinc ion fiber battery for electronic textiles. Sci Adv.

[109] Dong K, Peng X, Wang ZL (2020). Fiber/fabric-based piezoelectric and triboelectric nanogenerators for flexible/stretchable and wearable electronics and artificial intelligence. Adv Mater.

[110] He J, Lu C, Jiang H, Han F, Shi X, Wu J, Wang L, Chen T, Wang J, Zhang Y, Yang H, Zhang G, Sun X, Wang B, Chen P, Wang Y, Xia Y, Peng H (2021). Scalable production of high-performing woven lithium-ion fibre batteries. Nature.

[111] Ma X, Jiang Z, Lin Y (2021). Flexible energy storage devices for wearable bioelectronics. J Semicond.

